# Understanding molecular enzymology of porphyrin-binding α + β barrel proteins - One fold, multiple functions

**DOI:** 10.1016/j.bbapap.2020.140536

**Published:** 2020-09-04

**Authors:** Stefan Hofbauer, Vera Pfanzagl, Hanna Michlits, Daniel Schmidt, Christian Obinger, Paul G. Furtmüller

**Affiliations:** Department of Chemistry, Institute of Biochemistry, University of Natural Resources and Life Sciences, Vienna, Austria

**Keywords:** Ferrodoxin-like fold, Chlorite dismutases, Coproheme decarboxylases, Dye-decolorizing peroxidases

## Abstract

There is a high functional diversity within the structural superfamily of porphyrin-binding dimeric α + β barrel proteins. In this review we aim to analyze structural constraints of chlorite dismutases, dye-decolorizing peroxidases and coproheme decarboxylases in detail. We identify regions of structural variations within the highly conserved fold, which are most likely crucial for functional specificities. The loop linking the two ferredoxin-like domains within one subunit can be of different sequence lengths and can adopt various structural conformations, consequently defining the shape of the substrate channels and the respective active site architectures. The redox cofactor, heme *b* or coproheme, is oriented differently in either of the analyzed enzymes. By thoroughly dissecting available structures and discussing all available results in the context of the respective functional mechanisms of each of these redox-active enzymes, we highlight unsolved mechanistic questions in order to spark future research in this field.

## 1 Structural classifications and phylogeny of porphyrin-binding α + β barrel proteins

### 1.1 Structural classification

**Fig. 1:**
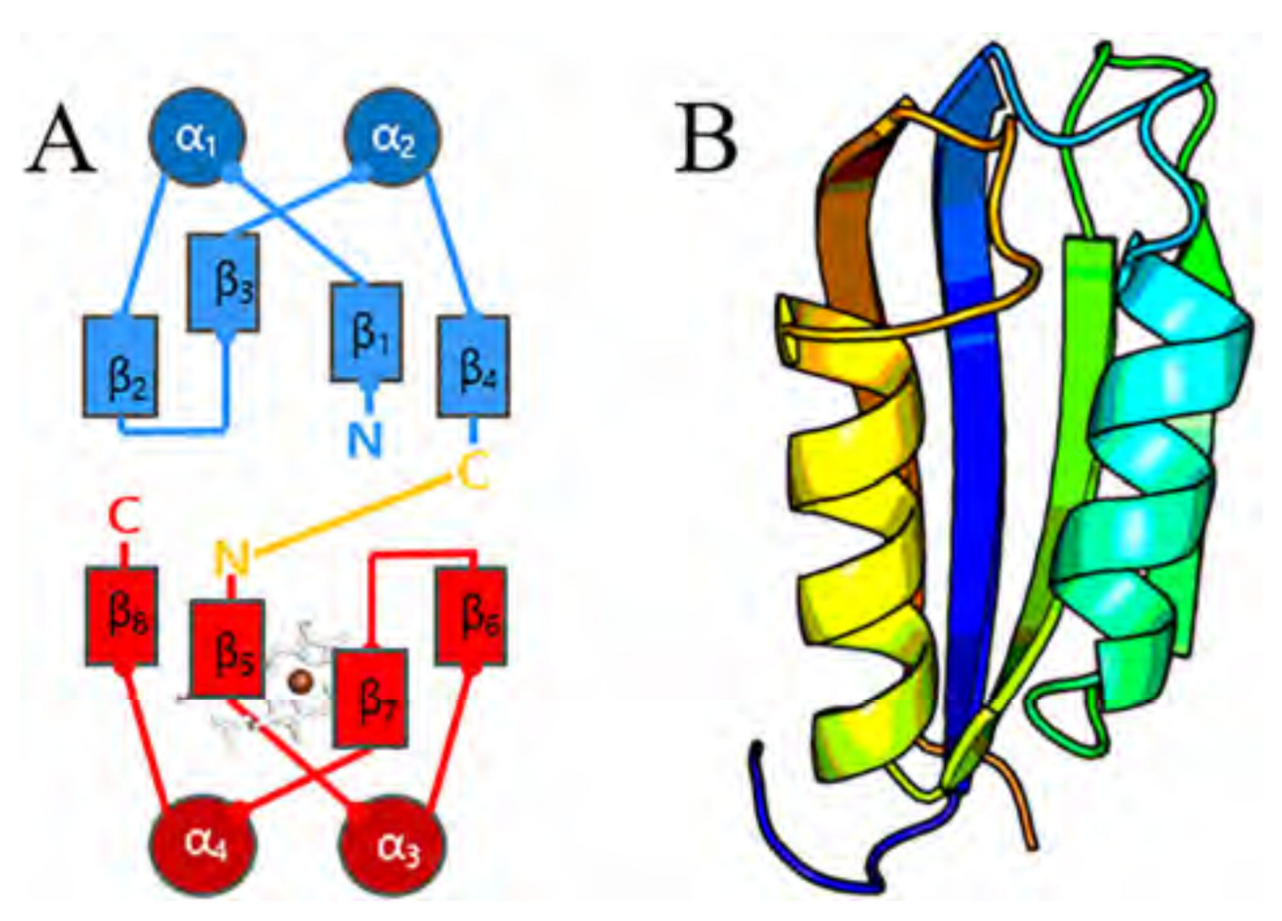
(A) Schematic representation of a subunit from the dimeric α + β barrel superfamily, consisting of two ferredoxin-like folds (N-terminal – blue, C-terminal – red, linker – yellow). (B) Example of a cartoon representation of one ferredoxin-like fold (taken from structure 2ACY); β_1_ – blue, α_1_ – cyan, β_2_ & β_3_ – green, α_2_ – yellow, β_4_ – orange. (For interpretation of the references to color in this figure legend, the reader is referred to the web version of this article.)

Irrespective of the system under investigation, building blocks of any kind need to be simple, robust, adaptable, and easily tunable for different purposes or specifications. In protein biochemistry, the smallest available building blocks are amino acids. These amino acids can form peptides or proteins, which exhibit secondary structures (e.g. α-helices, β-sheets, loops in all their variations), depending on the amino acid sequence (primary structure). A certain arrangement of secondary structures results in a fold (tertiary structure), like for example the **ferredoxin-like fold**, which consists of α-helical and β-sheet elements (Fig. 1).

In detail, it can be described as a long symmetrical hairpin with two terminal β-strands hydrogen-bonded to the central two β-strands, resulting in the formation of a four-stranded, antiparallel β-sheet covered on one side by two α-helices (Fig. 1B). The SCOP (Structural Classification of Proteins, http://scop.mrc-lmb.cam.ac.uk/scop/) server lists 65 “superfamilies” which utilize the **ferredoxin-like fold** (SCOP identifier 2000014). One of these is the **dimeric α + β barrel** superfamily (SCOP 3000089, InterPro Pfam CL0032), which consists of 23 families. As structure is more conserved than sequence, network analysis of these families reveal that this common core structure gave rise to a highly widespread functional landscape [1]. Out of the 23 structural families, four have been shown to be able to bind heme or heme derivatives (e.g. iron coproporphyrin III). These four families are (i) chlorite dismutases (Clds)/coproheme decarboxylases (ChdCs), (ii) dye-decolorizing-type peroxidases (DyPs)/EfeBs, (iii) aldoxime dehydratases (OxdAs), and (iv) IsdGs (heme degrading enzymes). These protein scaffolds employ two distinct packing topologies of the porphyrin molecule, which is manifested in the organization of the secondary structural elements [2]. It has to be noted that previously six families have been reported to bind porphyrins, but as is also evident from the network analysis by Celis & DuBois (2015) [1], Clds and ChdCs (formerly referred to as HemQs) as well as DyPs and EfeBs can be classified as one structural family, each. It is already evident from the nomenclature of the structural families that the performed functions of the respective enzymes/ proteins are highly diverse.

### 1.2 Phylogenetic and functional classification

Historically, the phylogenetic and structural relationships were not discovered and described in the way mentioned above. Indeed, with the discovery of the first Clds [3], DyPs [4] and EfeBs [5] respectively, separate phylogenetic trees were calculated for each enzyme class.

Phylogenetic analysis of putative chlorite dismutase sequences, identified two clades for the subgroup of active chlorite dismutases, both containing a conserved arginine residue on the distal heme side (discussion of the active site architecture will follow in a later section) [6,7]. These clades differ in sequence length (clade 1 is longer) and oligomerization status (clade 1, pentameric; clade 2, dimeric), as several biochemical studies on purified Clds from different organisms showed [8–15]. A large number of putative chlorite dismutase sequences was found to miss the catalytical distal arginine, which is essential for chlorite dismutase activity. These proteins were first described as chlorite dismutase-like proteins [7] and proved to be coproheme decarboxylases (formerly HemQs) a few years later [16–19]. ChdCs were further classified into four phylogenetically distinct clades [18,20]. The physiological role of Clds can only be speculated on, as they are able to efficiently reduce chlorite to chloride and thereby produce dioxygen. This can only be of use in perchlorate reducing bacteria [21,22], as chlorite occurs very rarely in the environment [23]. However, putative Cld sequences are also found in many other representatives of the bacterial world, such as nitrite-oxidizing bacteria and cyanobacteria. A possible role in nitrate respiration was indicated by a few studies [15,24]. The physiological role of coproheme decarboxylases on the other hand is well described for clades 1, 2, and 4, being responsible for the last step of heme biosynthesis in monoderm, and some diderm bacteria [16,18], which follow the so-called coproporphyrin-dependent heme biosynthesis pathway [20]. Structural and mechanistic details of Clds and ChdCs will be addressed in section 2, 3.1 and 3.3.

The first in-depth analysis of DyP sequences was performed in 2009, which led to the classification of type A, B, C and D of dye-decolorizing peroxidases [25]. Later type C and D were found to be one phylogenetic class; they are currently referred to as C/D or alternatively a different nomenclature was introduced, which categorizes DyPs in I (A), P (B), and *V* (C/D) [26]. Furthermore, sequence alignments of DyPs and EfeBs revealed that EfeBs are type A DyPs [27].

In most cases, the physiological functions of DyPs are completely unknown. DyPs from type C/D, which are exported and act extra-cellularly, can degrade lignin and do also occur in fungi [28,29]. A possible physiological role for the DyPC/D from *Bjerkandera adusta* is the degradation of antifungal anthraquinone compounds [30]. DyPs from type B are expressed intracellularly and no physiologically relevant function is assigned yet [31]. A proposed deferrochelatase activity of YfeX (DyPB of *Escherichia coli*) [32] was disproven [33]. Some type B DyPs, like the homologue from *Rhodococcus jostii*, are found in an open reading frame together with an encapsulating protein, which would enable them to be exported. This has led to the suggestion that they are also involved in lignin degradation [34–36]. Type A DyPs (EfeB in *E. coli*) were shown to be involved in the bacterial iron acquisition machinery as part of the EfeUOB transporter system in *E. coli*, but the exact role and the mechanism is unclear [37,38]. Type A DyPs have a tat signal [27,32] for export into the periplasm. As is evident from the previous lines, many different reactions for DyPs are under discussion. Their rather unfortunate name historically derives from the capability of mainly type C/D DyPs to degrade anthraquinone derivatives, such as Reactive Blue 5, as well as bulky compounds used in textile industry [4]. DyPs, in general, were found to have broad substrate specificity, as also artificial electron donors (e.g. ABTS, a classical peroxidase substrate) [39], non-phenolic lignin model compounds [29], azo-dyes [40], aromatic sulphides [41], manganese [42], and β-carotene [43] can be oxidized. Nevertheless, especially type B DyPs are very poorly performing peroxidases and oxidize substrates only at very low rates [31], sometimes comparable to the rates that free hemin produces in presence of hydrogen peroxide and a substrate [44]. Mechanistic aspects of the redox reactions performed by DyPs as well as detailed analysis of structural features of the active site will be addressed in section 2 and 3.2.

**Fig. 2:**
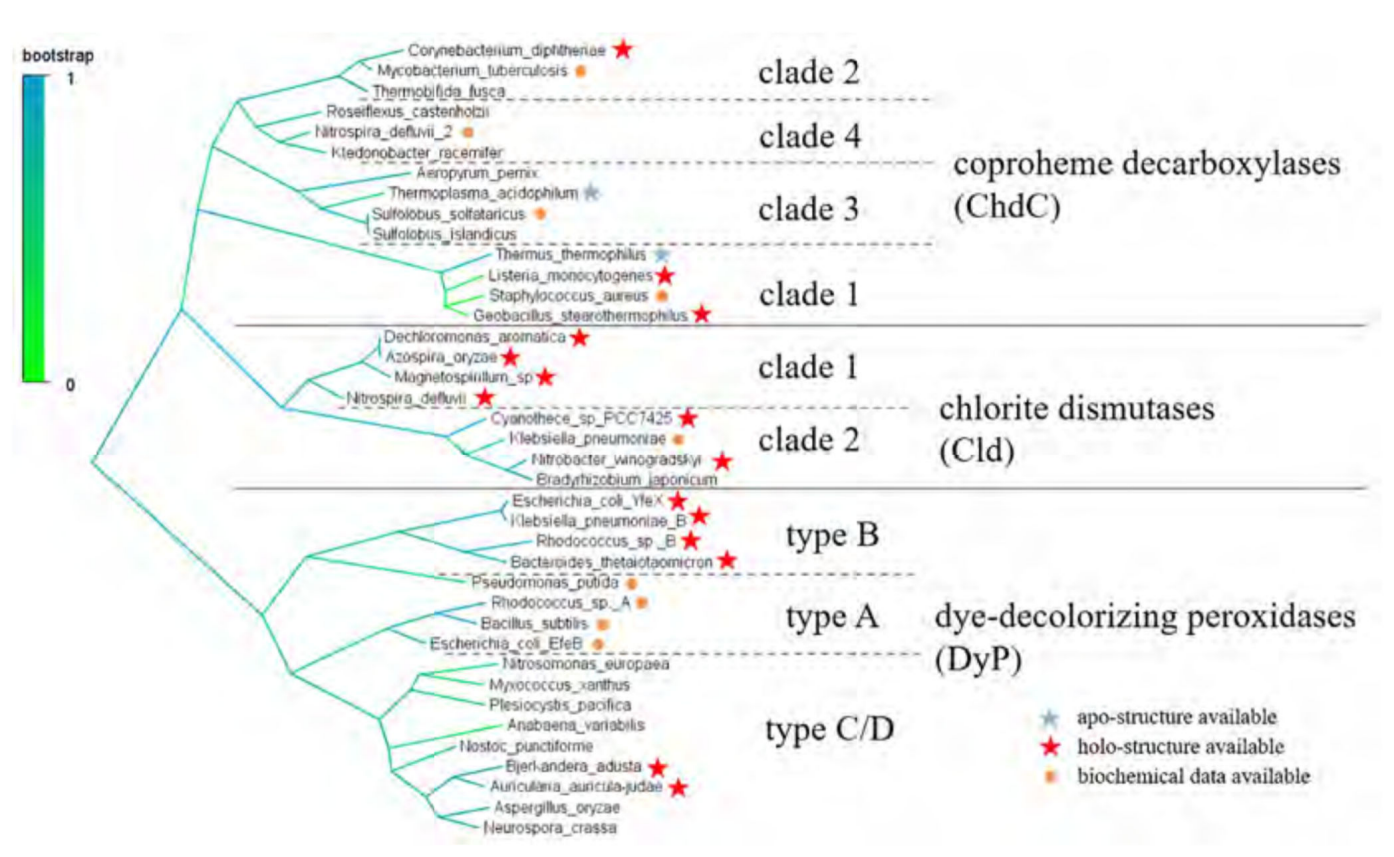
Maximum likelihood tree of selected representative ChdC, Cld, and DyP sequences.

The obvious structural similarity between Clds, ChdCs, DyPs, and EfeBs initially led to the description of the “CDE structural superfamily” (**C**ld, **D**yP, **E**feB) [45]. The calculation of maximum likelihood trees later showed that DyPs (including EfeBs) and Clds/ChdCs can be rooted against each other [46], as can be seen in the representative selection of Cld, ChdC and DyP sequences in Fig. 2.

Part of this structural superfamily forms the newest of four independently evolved peroxidase superfamilies, next to peroxidase-catalase, peroxidase-cyclooxygenase, and peroxidase-peroxygenase super-family. Originally it was referred to as “peroxidase-chlorite dismutase” superfamily [47], but as ChdCs are definitely not peroxidases, and Clds probably neither, this peroxidase superfamily currently consists only of DyPs. On the other hand also the nomenclature “CDE structural superfamily” is obsolete, as firstly EfeBs belong to DyPs and secondly and more importantly, all members of the former CDE superfamily are part of the larger **dimeric α + β barrel** superfamily (SCOP 3000089, Inter-Pro Pfam CL0032), introduced above, which also contains even more heme and/or porphyrin binding proteins (OxdA, IsdG) [1,2]. In this review, the focus will be on the dimeric α + β barrel enzymes **Clds, ChdCs** and **DyPs**, which utilize ferric heme or coproheme as a redox cofactor to yield their respective products. For these three enzymes, mechanistic similarities are present, as all three undergo an initial oxidization by an oxidant (hydrogen peroxide or chlorite). This distinguishes these three enzymes from OxdA and IsdG. In OxdA, the heme is in a ferrous resting state and does not need an oxidizing substrate to perform its reaction, which is the dehydration of aldoximes (R–CH=N–OH) to their corresponding nitriles (R-C ≡ N) [48–50]. IsdG is an enzyme that degrades heme in presence of O_2_, a reducing agent, and catalase [51–55].

## 2 Structure-function relationships of porphyrin-binding α + β barrel enzymes

### 2.1 Subunit structure

In-depth understanding of the driving forces of any catalytic activity of a given enzyme is achieved by critically and thoroughly studying structure and function of the target protein. Molecular enzymology aims at correlating structural and physical properties to explain distinct functions.

**Fig. 3:**
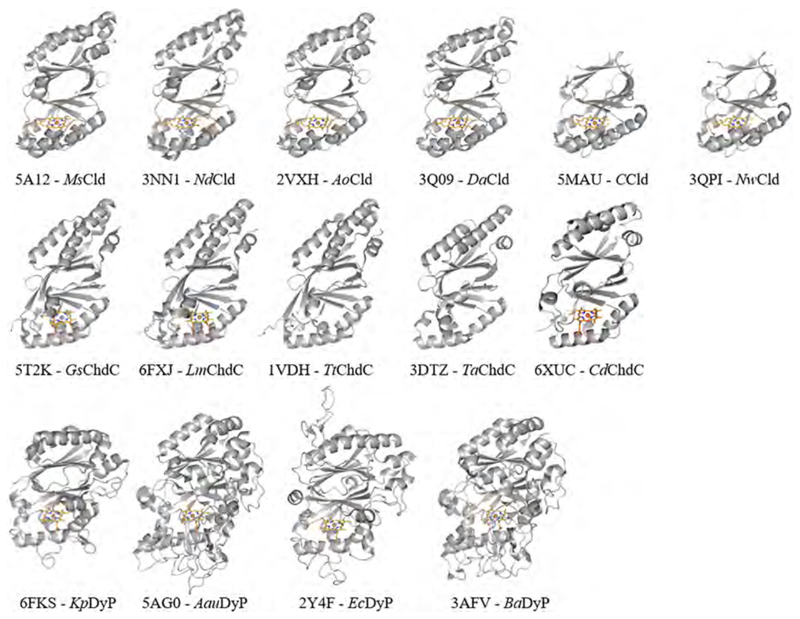
Subunit structure of representative Clds, ChdCs and DyPs. The backone is shown as grey cartoons and the heme or coproheme cofactor, if present is depicted as orange sticks. (For interpretation of the references to color in this figure legend, the reader is referred to the web version of this article.)

The message of the first chapter is that despite the functional diversity, chlorite dismutases, dye-decolorizing peroxidases and coproheme decarboxylases all belong to the same structural superfamily. This is evident by visualizing the single subunits of the discussed enzymes (Fig. 3). There are approximately 20 wild-type and variant chlorite dismutase structures from six different organisms available in the protein data bank (www.pdb.org). In Fig. 3, subunits from Clds of *Magnetospirillum sp.* [56], *Nitrospira defluvii* [7], *Azospira oryzae* [57], *Dechloromonas aromatica* [14], *Cyanothece sp.* PCC7425 [58], and *Nitrobacter winogradskyi* [9] are presented. Clade 1 Cld subunits consist of two ferredoxin-like folds, of which the C-terminal fold is able to bind heme. Clade 2 Clds lack the N-terminal α-helices, making them considerably shorter than Clade 1 Clds. Coproheme decarboxylases show a subunit architecture highly similar to Clade 1 Clds, with the coproheme binding site in the C-terminal ferredoxin-like fold. ChdC structures of five organisms are deposited in the pdb; from *Geobacillus stearothermophilus* [59], *Listeria monocytogenes* [60–62], *Thermus thermophilus* [63], *Thermoplasma acidophilum* (PDB ID 3DTZ) and, *Corynebacterium diphteriae* [64]. Structural information on DyPs is present for even more representatives, as approximately 50 structures from 16 different organisms can be found in the pdb. In Fig. 3, four representative structures are depicted; type B DyP of *Klebsiella pneumoniae* [31], type C/D DyP of *Auricularia auricular judae* [65] and *Bjerkandera adusta* [66] and type A DyP (EfeB) of *Escherichia coli* (PDB ID 2Y4F).

Although DyPs overall have longer protein sequences, two ferredoxin-like folds are arranged similar to Clds and ChdCs. Instead a high number of long flexible loops bridge the α-helices and β-sheets in DyP structures (Fig. 3). The heme binding site is again located in the C-terminal part of the subunit. The extensive presence of surface defining loops in type C/D DyPs (Fig. 3) is crucial for the interaction with potentially large substrates and the establishment of long range electron transfer, which is necessary to oxidize e.g. bulky anthraquinone dyes [67]. Type B DyP subunits are the most compact.

### 2.2 Oligomeric structure

**Fig. 4:**
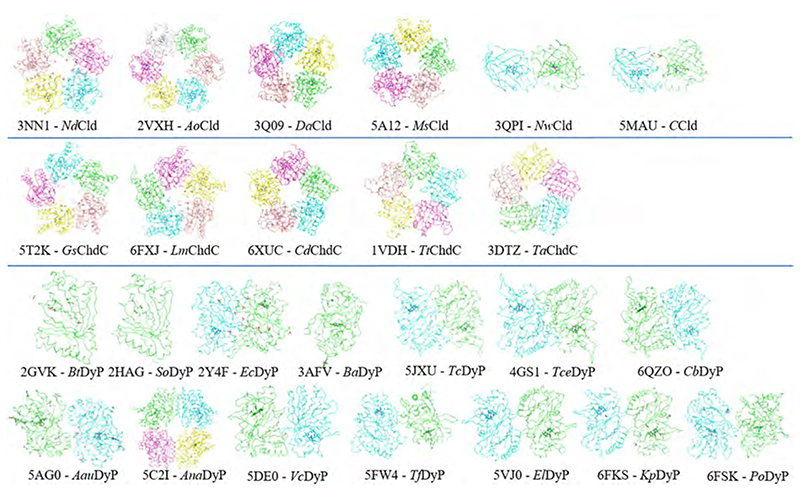
Ribbon representation of oligomeric assemblies of representative crystal structures of Clds, ChdCs and DyPs. Cld structures from six different organisms, ChdCs structures from five different organisms and DyP structures from 14 organisms are presented.

The oligomeric assembly of Clds, ChdCs and DyPs can range from monomeric to hexameric complexes. Clade 1 Clds and ChdCs crystallize predominantly as pentamers, with the exception of *Ao*Cld (hexamer) [57] (Fig. 4). Before the first crystal structures were solved, Clds from different organisms were believed to be tetramers in solution, but also pentamers were reported [12,14,57,68]. This diversity in reported oligomerization is potentially due to buffer conditions or methodological inaccuracies. Clade 2 Clds always form dimers, also in solution, as confirmed by multi-angle laser light scattering [15,69]. DyPs exhibit the highest variability in oligomeric assemblies, as they can occur as monomers (*Bacteroides thetaiotaomicron* or *Shewanella oneidensis* DyPs) [70], head to tail dimers (most commonly observed) [31,44,65,66,71–75] or tetramers (*Anabaena* sp. DyP) [76].

In all cases the purpose of the respective oligomerization can only be speculated on. For instance, as the physiological substrates of DyPs are not known in most cases, no conclusions can be derived on the necessity of the high oligomeric diversity. The necessity for assembly in pentamers is also unknown for ChdCs but a regulatory mechanism can be expected due to the apparent cooperativity which is shown during heme binding [60]. ChdC’s substrate, coproheme, is produced by co-proporphyrin ferrochelatase (CpfC), which is a monomeric enzyme [77].

Studies focusing on the protein-protein interaction will prove highly valuable in understanding the substrate and product trafficking within the coproporphyrin-dependent heme biosynthesis pathway, which utilizes ChdC. One study reports a cross-talk between CpfC, ChdC and the dimeric protein IsdG [78], which belongs to the same structural superfamily (SCOP 3000089, InterPro Pfam CL0032), and is involved in bacterial heme degradation to obtain iron [79].

The functional importance of the oligomerization of Clds cannot be answered, as ligand binding studies and steady-state kinetic experiments do not show cooperativity [80,81]. Further, direct comparison of a dimeric Clade 2 Cld with a pentameric Clade 1 Cld proved that the redox thermodynamics, which govern the catalytic potential of an enzyme, were almost identical [11] but that the thermal and conformational stability was significantly altered, as the pentameric enzyme was much more stable than the dimeric one [8,10]. Interface analysis of pentameric Clds using the PDBePISA server show that many non-covalent interactions are established (18.5 hydrogen bonds and 4.5 salt bridges on average), especially in the non-heme binding N-terminal ferredoxin-like fold, which is missing in Clade 2 Clds. On the other hand, a pentameric assembly does not guarantee high thermal and conformational stability, as coproheme-*Lm*ChdC has a significantly lower thermal stability (*T*_M_ = 55 °C) than pentameric *Staphylococcus aureus* ChdC (*Sa*ChdC) (*T*_M_ = 73 °C) [82] or pentameric *Nd*Cld (*T*_M_ = 91 °C) [8]. Interestingly, chlorite dismutase from *Dechloromonas aromatica* appears to lose its oligomerization upon exchange of a conserved tryptophan residue, which is located in the C-terminal ferredoxin-like fold in close proximity to the heme cofactor and at the subunit interface. The corresponding *Da*Cld tryptophan *v*ariant did not show any chlorite degrading activity [83].

### 2.3 Binding orientation of the cofactor

**Fig. 5:**
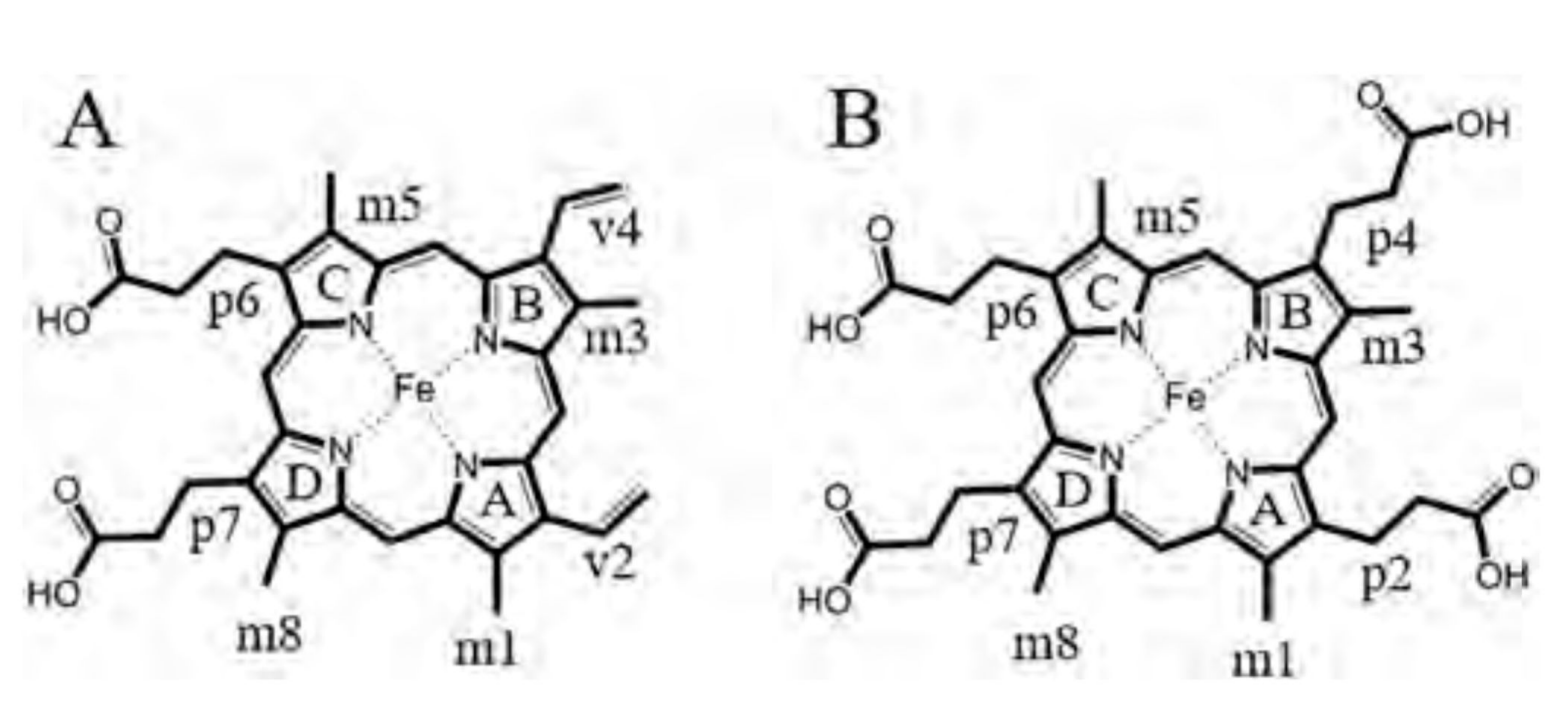
Chemical structures of heme *b* (A) and coproheme (B).

In all three protein families, the respective iron containing porphyrin is bound to the C-terminal ferredoxin-like fold in the exact same binding cleft between the β_5_ and β_7_ sheet of the secondary structure (Figs. 1A, 3). The orientation of heme *b* or coproheme in the active site differs substantially between the three protein families. Both heme *b* (Fig. 5A) and coproheme (Fig. 5B) are asymmetric porphyrins with iron as the central ligated metal. In order to assess the orientation within the protein environment, the numbering of pyrrole rings (A, B, C, and D) and methyl (m), vinyl (v) and propionyl (p) substituents (1–8) are shown in Fig. 5A and B.

**Fig. 6:**
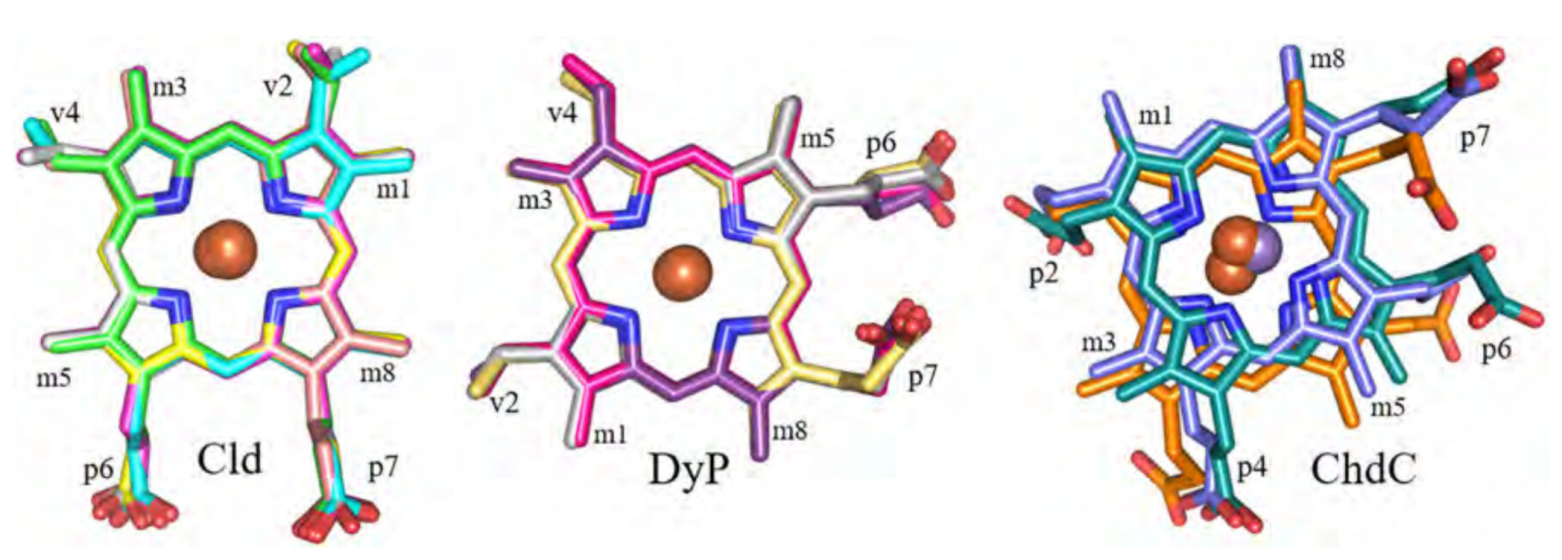
Orientation of the heme *b* or coproheme within the respective Cld-, DyP-, and ChdC- subunit. All subunits were structurally aligned. Porphyrins of respective proteins are colored as following: *Ms*Cld (green, 5A12), *Nd*Cld (cyan, 3NN1), *Ao*Cld (magenta, 2VXH), *C*Cld (yellow, 5MAU), *Nw*Cld (salmon, 3QPI), *Da*Cld (light grey, 3Q09), *Kp*DyP (hotpink, 6FKS), *Aau*DyP (sand, 5AG0), *Ec*DyP (purple, 2Y4F), *Ba*DyP (grey, 3AFV), *Lm*ChdC (blue, 6FXJ), *Gs*ChdC (turquoise, 5T2K), *Cd*ChdC (orange, 6XUC). (For interpretation of the references to color in this figure legend, the reader is referred to the web version of this article.)

Structural alignment of six Clds, four DyPs and three ChdCs, all containing a heme *b* or coproheme in the active site, shows that the binding orientation differs from each protein family to the other. The solvent exposed surface of the respective subunit is always on the right in the representation of the porphyrins (Fig. 6). In Clds, the heme *b* of each displayed subunit overlays nicely and only small variations in the orientations of the propionate and vinyl groups are visible. This can be attributed to some flexibility, but also in part to poor electron densities of these atoms in the models of the X-ray crystal structures.

Heme *b* in DyPs is orientated not only 90° rotated compared to heme *b* in Clds, but also 180° rotated in the x-axis (as presented in Fig. 6). This is manifested by the position of the vinyls and propionate groups, which are, as in Clds, the only observed flexible parts of the heme *b* structure. In DyPs the substrate access channel is much narrower than in Clds (see below), and the charged propionate groups point towards the protein surface, whereas in Clds methyl groups 1 and 8 are surface exposed.

Structures of coproheme bound ChdCs show the highest flexibility in the binding pocket of the C-terminal ferredoxin-like fold. The orientation itself is identical in all three coproheme bound solved structures, which is 90° rotated to the orientation of heme *b* in Clds. This was not clear from the first solved structure of coproheme-*Lm*ChdC (Firmicutes) [61]. Here the coproheme was initially placed in the same orientation as in Clds, only two of the four propionates of coproheme were well resolved. Later, a structure of *Gs*ChdC with a manganese coproheme, which is inactive towards hydrogen peroxide, revealed the correct orientation [59], and another, cyanide inhibited structure of iron coproheme of *Lm*ChdC confirmed this binding orientation in the resting state [62]. Inactivation/inhibition of the enzyme is required to obtain the resting state as autoactivation of dissolved oxygen leads to formation of hydrogen peroxide and consequently to a slow conversion of coproheme to the three-propionate reaction intermediate. Alternatively, crystallization trials with deoxygenized buffers under anaerobic conditions might be possible to obtain the same structures, however, nothing in this respect has been reported to date.

With the additional data in hand, the first solved structure of coproheme-*Lm*ChdC was later revisited and re-refined. In combination with extensive in-solution resonance Raman spectroscopic studies during turnover, at room and low (100 K) temperatures, it was shown that the three-propionate intermediate rearranges during catalysis within ChdC’s active site. The product, heme *b*, thus eventually is oriented as in Clds [62]. The recently solved structures of coproheme in ChdC of the actinobacterium *C. diphteriae* agrees with the current hypothesis of the binding orientation in the resting state and the rearrangement of the three-propionate intermediate during turnover. These structures are solved to a higher resolution and do not leave any room for misinterpretation [64]. To date, the orientation of the product heme *b* within the active site of ChdCs is unknown. For firmicute representatives spectroscopic studies on the environment of propionates p6 and p7 indicate an orientation similar to the position of heme *b* in Clds [19,62]. This question will be crucial for future studies, as also the transport mechanism of the product (heme *b*) to various apo-proteins is yet unknown.

### 2.4 Substrate access channels

**Fig. 7:**
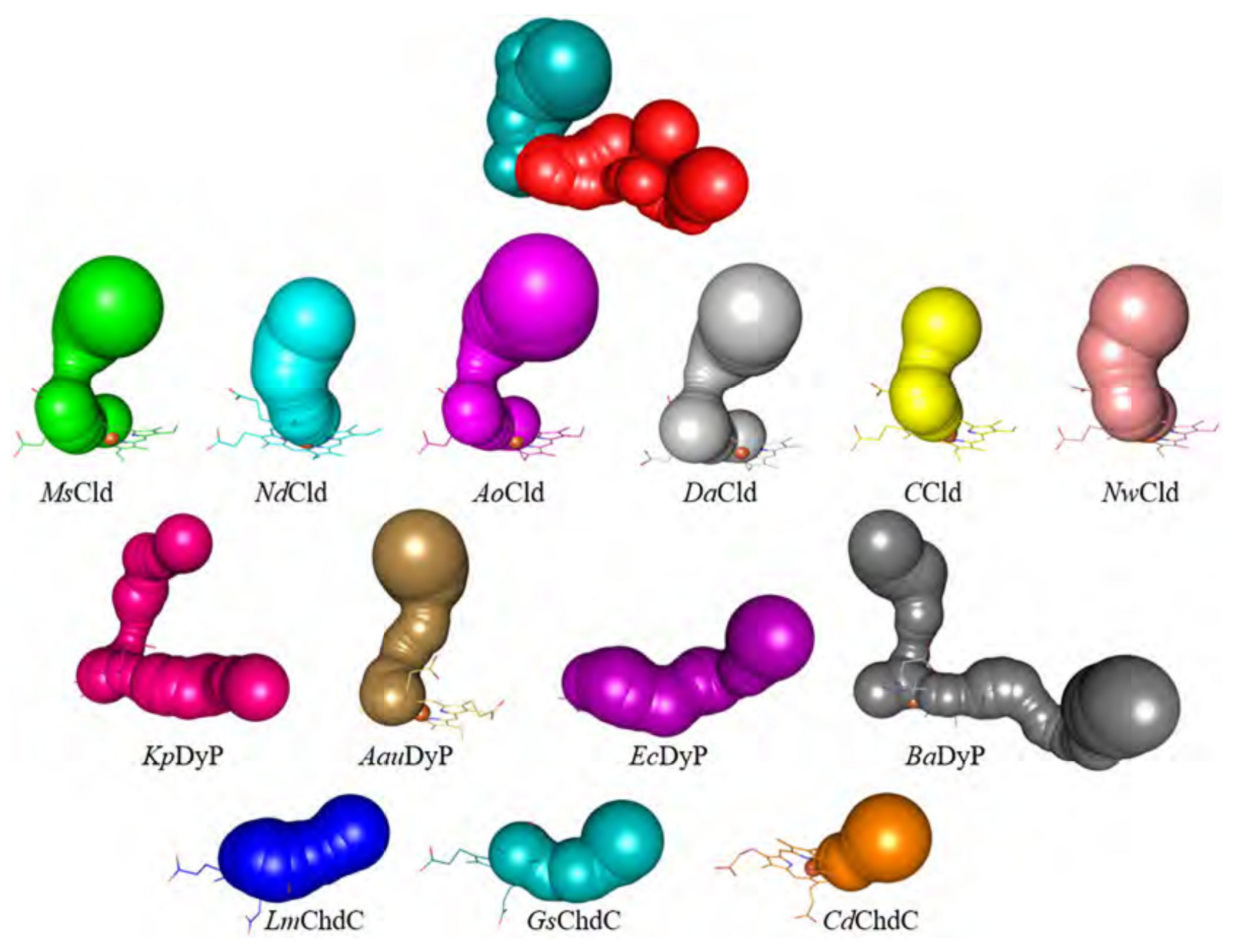
Substrate access channels (represented as colored spheres). All subunits were structurally aligned. At the top, an overlay of all calculated substrate channels is presented. Two predominant pathways were observed: “deepteal” and “red”. Calculations with CAVER 3.0 were performed with the following settings: Minimum probe radius = 1.2; Shell depth = 8; Shell radius = 7; Clustering threshold = 4; Input model: always only all respective amino acids; Starting point was always specified as the central porphyrin metal; Starting point optimization - maximum distance = 3; desired radius = 5. Channels within the respective proteins are colored as following: *Ms*Cld (green, 5A12), *Nd*Cld (cyan, 3NN1), *Ao*Cld (magenta, 2VXH), *C*Cld (yellow, 5MAU), *Nw*Cld (salmon, 3QPI), *Da*Cld (light grey, 3Q09), *Kp*DyP (hotpink, 6FKS), *Aau*DyP (sand, 5AG0), *Ec*DyP (purple, 2Y4F), *Ba*DyP (grey, 3AFV), *Lm*ChdC (blue, 6FXJ), *Gs*ChdC (turquoise, 5T2K), *Cd*ChdC (orange, 6XUC). (For interpretation of the references to color in this figure legend, the reader is referred to the web version of this article.)

The active site is the place where chemistry/catalysis happens. Therefore, the shape and characteristics of the substrate access channel, which connects the solvent space at the protein surface with the heme *b* cofactor or coproheme in the active site, is of utmost importance. Calculations using CAVER 3.0 [84] give a good impression and overview of representative substrate access channels in Clds, DyPs, and ChdCs. In principle, the porphyrin cofactor in the dimeric α + β barrel proteins has two main routes towards the protein surface. One is depicted in deepteal (dark cyan) on the top of Fig. 7, the other one in red. In all Clds the most prominent access route reaches the distal heme side from the top (route “deepteal”, Fig. 7), whereas all ChdCs have their main substrate channel access towards the propionates, from the side (route “red”, Fig. 7). DyPs appear to be very adaptable and can either have two main substrate access channels of similar importance (routes “deepteal” and “red”: *Kp*DyP, *Ba*DyP), or a single access channel (route “deepteal”: *Aau*DyP; route “red”: *Ec*DyP). Depending on the settings of the CAVER calculations, a second narrower access channel in B-type *Ec*DyP is present (corresponding to the route “deepteal”), which most probably is the access path for hydrogen peroxide. This indicates that the geometry of the substrate channels differs in DyPs between phylo-genetically distinct classes and may be partially responsible for differential substrate specificity.

**Fig. 8:**
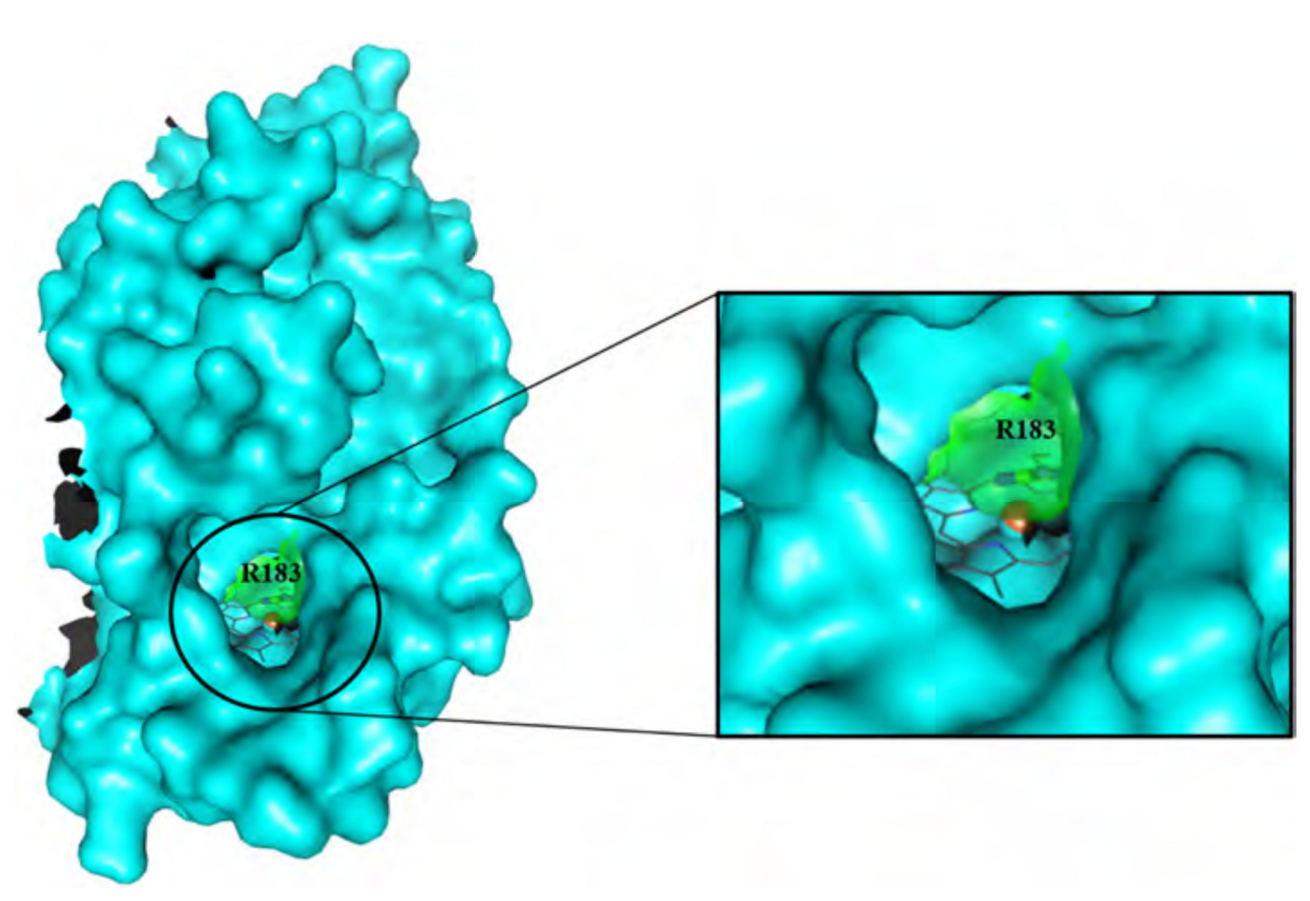
Surface representation of Chain A of *Nd*Cld (cyan) and Arg183 of *Ms*Cld (sticks and semi-transparent surface; green). On the right, a close-up of the substrate access channel is shown. The heme *b* cofactor is depicted in dark grey. (For interpretation of the references to color in this figure legend, the reader is referred to the web version of this article.)

Interestingly, the calculated bottleneck radii within Clds vary between 1.6 Å (*Ms*Cld) and 2.8 Å (*Nd*Cld), while still occupying the same general direction. Bottleneck radii of *Ao*Cld (1.7 Å) and *Da*Cld (1.7 Å) cluster with *Ms*Cld while *C*Cld (2.2 Å) and *Nw*Cld (2.6 Å) cluster with *Nd*Cld. This clustering reflects the position of the catalytically relevant distal arginine residue of Clds in the crystallographic model, but is not related to the phylogenetic clades. The mechanistic implications of this residue and its flexibility will be the focus of section 3.2. Here, we see that the distal arginine governs the bottleneck radius of the most probable access channel in Clds. In the *Nd*Cld, *C*Cld, and *Nw*Cld channel-cluster the arginine is modelled in an “out”-position, whereas in the other three structures (*Ao*Cld, *Ms*Cld, *Da*Cld) it is found in an “in”-position. To visualize the effect on the channel width, Fig. 8 shows the surface of the *Nd*Cld subunit A in cyan, a representative heme *b* in dark grey in an overlay with Arg183 (“in” conformation) of *Ms*Cld as a stick representation (green) together with its surface (semi-transparent green). The substrate access channel is wide open when only the *Nd*Cld surface is considered, while the inward oriented arginine in *Ms*Cld lowers this to nearly half of the initial diameter (Fig. 8).

**Fig. 9:**
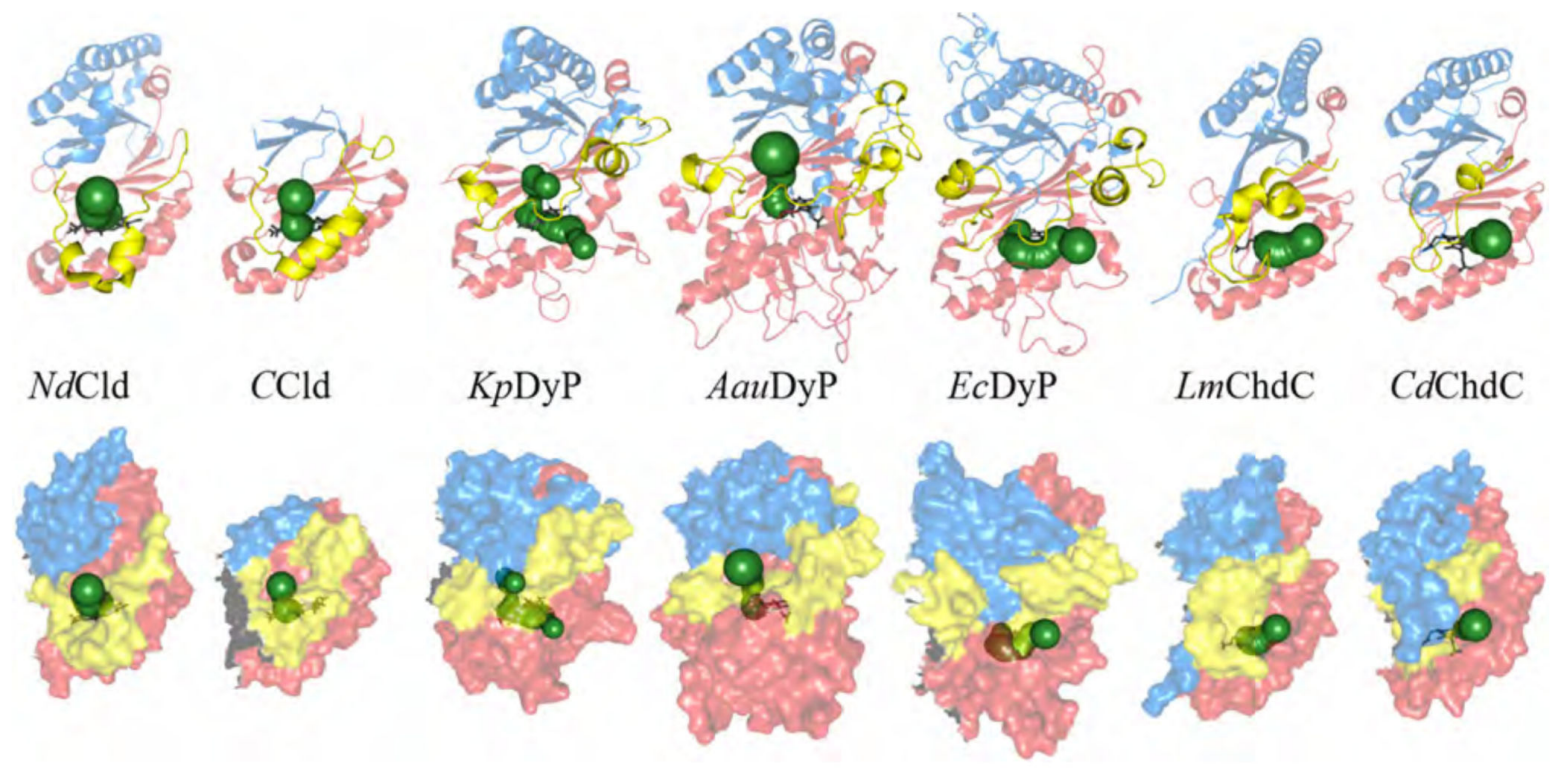
Conformation of the linker loop between the two ferredoxin-like subdomains of representative Cld, DyP, and ChdC subunits. The N-terminal ferredoxin-like fold is depicted in blue, the C-terminal heme *b* or coproheme binding ferredoxin-like fold is shown in red. The linker, connecting both ferredoxin-like folds is shown in yellow. Calculated substrate channels are represented as green spheres. The top row shows the secondary structural elements as cartoons, in the bottom row the surface is presented in the respective colors. The heme *b* or coproheme cofactors are depicted in dark grey. (For interpretation of the references to color in this figure legend, the reader is referred to the web version of this article.)

Whether a substrate access channel in one of the discussed enzymes follows the “deepteal” path or the “red” path is determined by the shape of a single flexible loop (yellow in Figs. 1A and 9). This loop connects the N-terminal and the C-terminal ferredoxin-like folds, being the linker between the β4 and β5-sheets [17,18]. In Clds this loop forms the bottom of the access channel, whereas in ChdCs the access channel is flanked by this loop (Fig. 9). Homology modelling of *Cd*ChdC, which was performed before the experimental 3D-structure was solved, incorrectly suggested that the linker loop was forming an access channel similarly shaped as in pentameric Class I Clds [18], because these Cld structures were chosen by the modelling software as the most suitable templates. In the now available high-resolution crystal structure (6XUC) the loop adapts a conformation similar to the one of *Lm*ChdC, though significantly shorter (Fig. 9). Also, the catalytically important His118 residue, which is responsible for deprotonation of an incoming hydrogen peroxide prior to Compound I formation, is located on this loop [64]. In DyPs, the catalytically relevant distal aspartate, whose purpose is also to facilitate deprotonation of the hydrogen peroxide in Compound I formation, is located on the linker between the β_4_ and β_5_-sheets as well [31,66,85]. This emphasizes once again that evolution uses this linker loop between the two ferredoxin-like folds to modify substrate specificity and optimize enzymatic properties.

### 2.5 Active site architecture

#### 2.5.1 Proximal side

**Fig. 10:**
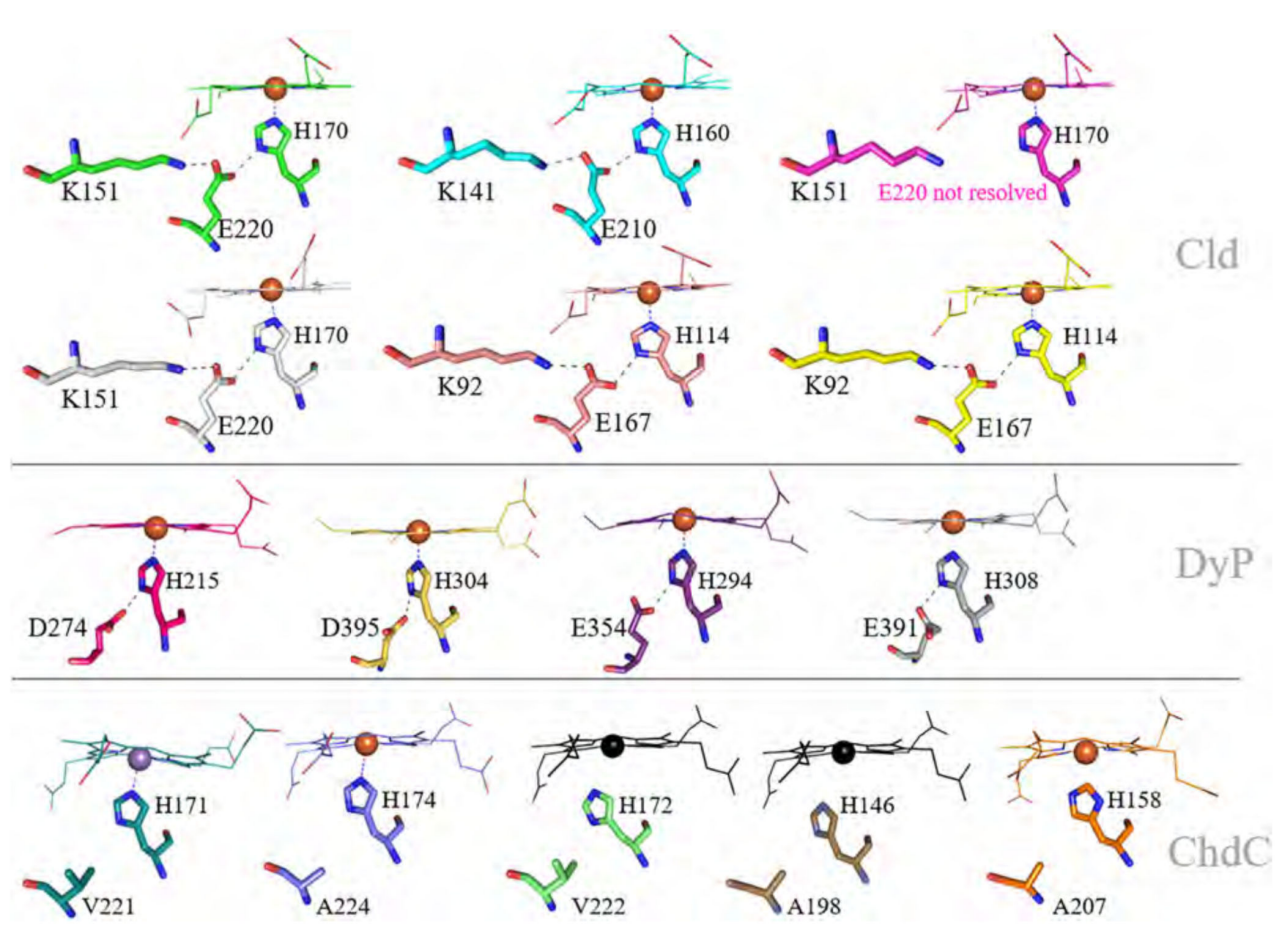
Proximal hydrogen bonding network of Clds, DyPs and the proximal environment of ChdCs. In the top two rows, Clds are presented in green (*Ms*Cld, 5A12), cyan (*Nd*Cld, 3NN1), magenta (*Ao*Cld, 2VXH), yellow (*C*Cld, 5MAU), salmon (*Nw*Cld, 3QPI), light grey (*Da*Cld 3Q09). DyPs are colored in hotpink (*Kp*DyP, 6FKS), sand (*Aau*DyP, 5AG0), purple (*Ec*DyP, 2Y4F), grey (*Ba*DyP, 3AFV). In the bottom row ChdCs are represented in turquoise (*Gs*ChdC, 5T2K), blue (*Lm*ChdC, 6FXJ), orange (*Cd*ChdC, 6XUC), lightgreen (*Tt*ChdC, 1VDH) and dark orange (*Ta*ChdC, 3DTZ). For better orientation, the coproheme of the *Lm*ChdC structure was placed in apo-structures of *Ta*ChdC and *Tt*ChdC in black. (For interpretation of the references to color in this figure legend, the reader is referred to the web version of this article.)

**Fig. 11:**
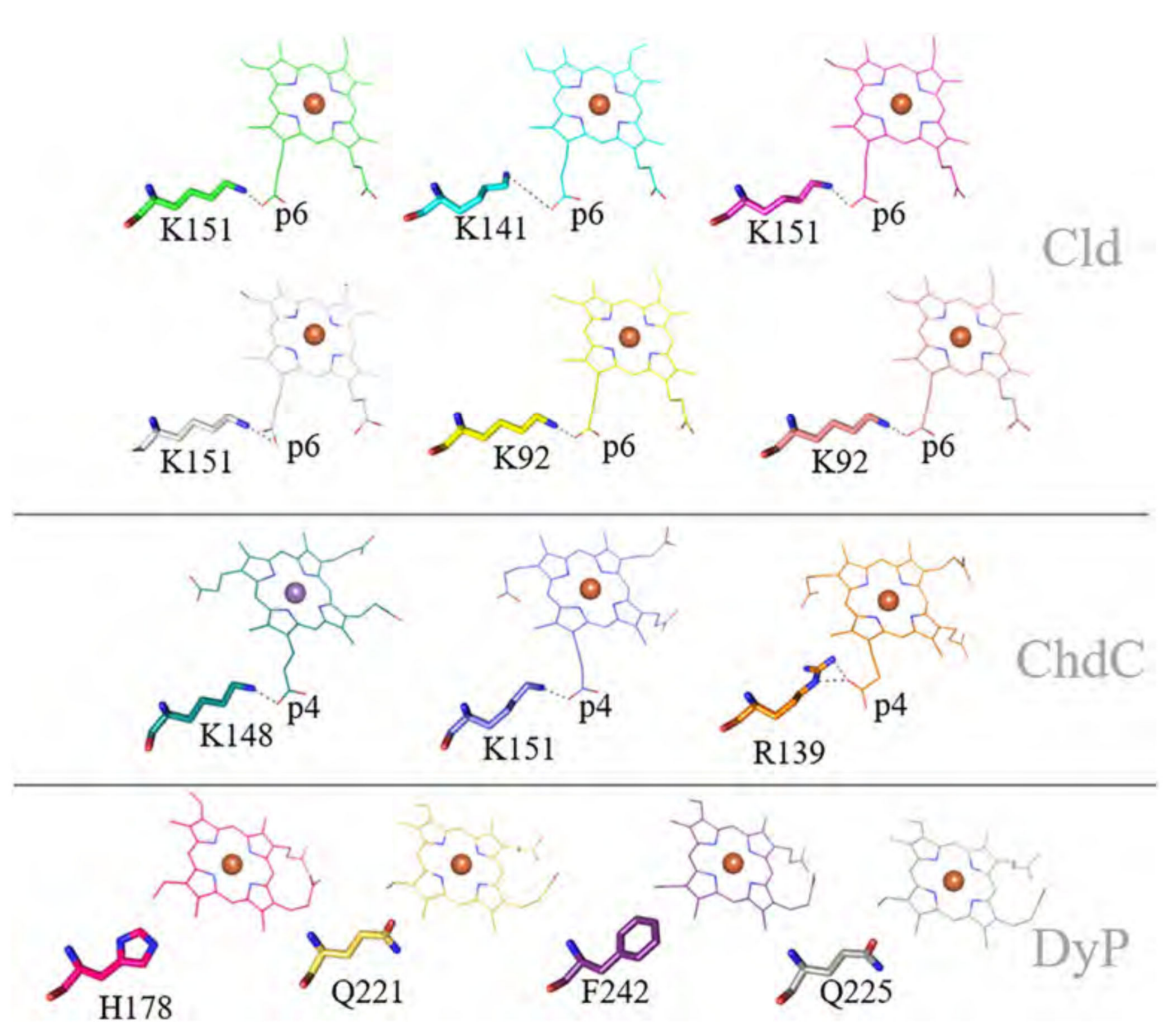
Structural comparison of the lysine/arginine position conserved in Clds and ChdCs, which is a histidine, phenylalanine or glutamine in DyPs. Green (*Ms*Cld, 5A12), cyan (*Nd*Cld, 3NN1), magenta (*Ao*Cld, 2VXH), light grey (*Da*Cld 3Q09), yellow (*C*Cld, 5MAU), salmon (*Nw*Cld, 3QPI), turquoise (*Gs*ChdC, 5T2K), blue (*Lm*ChdC, 6FXJ), orange (*Cđ*ChdC, 6XUC), hotpink (*Kp*DyP, 6FKS), sand (*Aau*DyP, 5AG0), purple (*Ec*DyP, 2Y4F), grey (*Ba*DyP, 3AFV). (For interpretation of the references to color in this figure legend, the reader is referred to the web version of this article.)

The central iron of the heme *b* or coproheme is in all three enzymes coordinated on the proximal side by a histidine residue. The proximal side is further involved in extensive hydrogen bonding networks. The imidazolate character of the proximal histidine is enhanced in Clds and DyPs by hydrogen bonds to glutamate (Clds) and glutamate or aspartate (DyPs). In ChdCs no conserved hydrogen-bonding partner is present. The relevant position is instead occupied by an alanine or valine (Fig. 10). The proximal hydrogen bonding network in Clds extends to a lysine residue, which additionally hydrogen bonds with propionate 6 (p6) (Fig. 11). In ChdCs, this lysine (or arginine in actinobacterial representatives) is also present and coordinates – due to the rotated orientation of coproheme-propionate 4 (p4) as shown in Fig. 6. In DyPs vinyl 4 (v4) occupies this position instead of p6, as heme *b* is rotated by 90° compared to Clds. Vinyl 4 does not require coordination by a positively charged residue and consequently no lysine or arginine is found in DyPs at this site (Fig. 11).

The proximal hydrogen bonding network modulates the imidazolate character of the coordinating histidine and consequently also the redox potential (reviewed in detail in reference [86]) and the positional integrity of the incorporated heme species. The imidazolate character of the proximal histidine can be determined by the examination of the *ν*(Fe-His) stretching by resonance Raman spectroscopy of the ferrous heme protein, when the 441.6 nm line of a He–Cd laser is used for enhancement. The *ν*(Fe-His) stretching band exhibits a wavenumber between 205 and 250 cm^−1^. When the proximal histidine is strongly interacting with other residues, the imidazolate character increases and the *ν*(Fe-His) stretching band is between 240 and 250 cm^−1^ [87]. A weak imidazolate character, on the other hand, is characterized by frequencies of 205 to 215 cm^−1^. Studies investigating the imidazolate character of the proximal histidine are available for the DyP of *Vibrio cholerae*, which has a *ν*(Fe-His) of 229 cm^−1^ [71], similar to the ones determined for various pentameric and dimeric Clds; Cld from *Dechloromonas aromatica* 222–224 cm^−1^ [13], Cld from *Nitrospira defluvii* 226 cm^−1^ [88], Cld from *Klebsiella pneumoniae* 229 cm^−1^ [15], Cld from *Cyanothece* sp. PCC7425 231 cm^−1^ [58]. All these frequencies indicate a pronounced imidazolate character possibly due to the H-bonding interaction with a negatively charged residue (aspartate or glutamate).

A study on *Nd*Cld targeted the proximal hydrogen bonding network by exchanging the glutamate (E210A), H-bonded to the proximal histidine, and lysine (K141E), which establishes an H-bond to this glutamate. Removing the glutamate lowers the frequency by 10 cm^−1^ to 216 cm^−1^ and an exchange of the lysine led to a frequency at 221 cm^−1^ [88]. In *Nd*Cld, the proximal H-bonding network is highly important for the integrity and stability of the heme within the active site. This is demonstrated by the fact that, in contrast to wild-type *Nd*Cld, heme can be extracted from the proximal mutants (K141E, E210A), by apomyoglobin [88].

Similarly, heme *b* can be extracted from ChdC from *Listeria monocytogenes*, whose distal H-bonding network was mimicked in the *Nd*Cld variant E210A [60]. This data is also in agreement with the observed *ν*(Fe-His) stretching frequency of 214 cm^−1^ for *Lm*ChdC [61] and 213 cm^−1^ for *Sa*ChdC [19], which is well comparable to the 216 cm^−1^ of *Nd*Cld E210A [88].

#### 2.5.2 Distal side

In principle, catalysis happens at the distal side of the heme. This is where the substrate channels lead to. Further, the active site architecture on this side determines the ability to bind and turnover substrates like hydrogen peroxide and chlorite. The heme *b* or coproheme iron in Clds, DyPs and ChdCs is always coordinated by four nitrogen atoms of the porphyrin macrocycle and one nitrogen of the proximal histidine. The sixth coordination site can remain unoccupied, leading to a five-coordinated state; it can also be occupied by several high-spin (e.g. fluoride, chloride) or low-spin ligands (e.g. cyanide, imidazole, hydroxide) resulting in a six-coordinated species.

While the electronic mechanisms of the redox reactions and the importance of the catalytically relevant residues for each enzyme class will be discussed in a separate section, the structural constraints and features of the catalytically relevant residues are described in this chapter.

In Clds, the catalytically relevant residue is a distal arginine [7,9,15,80,81], which is also found to be important in DyPs [85]. DyPs additionally possess a catalytically relevant distal aspartate [39,89], which is part of the flexible loop connecting the N- and the C-terminal domain, described above (Fig. 9). In ChdCs, a tyrosine at the edge of the distal pocket, in close proximity to propionate 2 (p2) in the resting state is essential for catalysis [62,90,91]. For ChdCs from Actinobacteria, a distal histidine residue was identified to be important for deprotonation of the incoming hydrogen peroxide, which is part of the same flexible loop described above [64]. In the following figures of the active site structures, amino acid side chains at the positions of the catalytically relevant residues, and also the proximal histidine are presented.

**Fig. 12:**
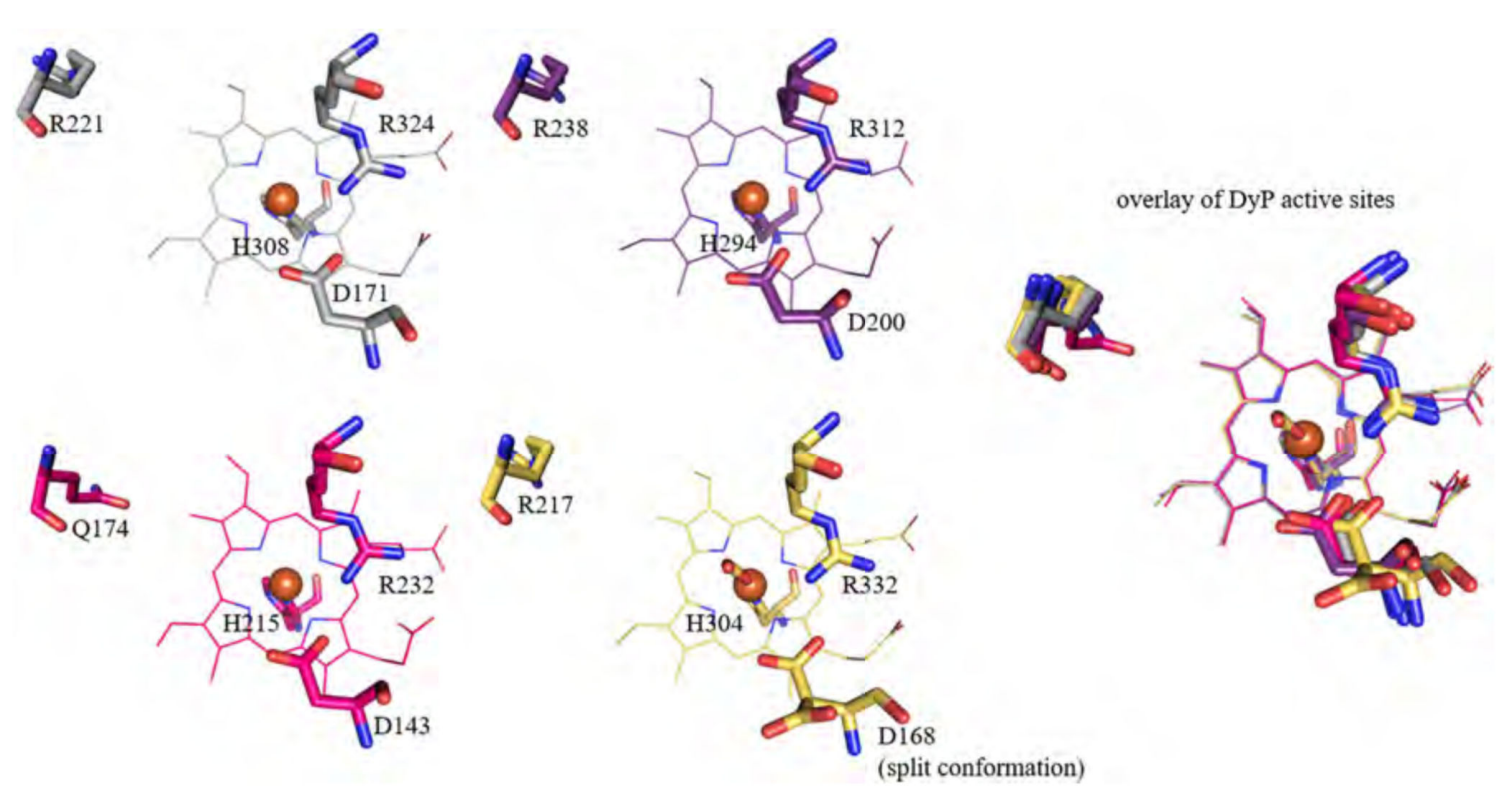
Distal side architecture of DyPs. Relevant active site residues and heme molecules are shown in grey (*Ba*DyP, 3AFV), purple (*Ec*DyP, 2Y4F), hotpink (*Kp*DyP, 6FKS) and sand (*Aau*DyP, 5AG0). On the right an overlay of the four representative DyP structures is presented. (For interpretation of the references to color in this figure legend, the reader is referred to the web version of this article.)

In DyPs, the structural flexibility within the active site is minimal. All structures show the active site arginine, which is H-bonded to propionate 7 (p7), in the same orientation. Similarly, the position and orientation of the distal aspartate is just slightly altered in the presented structures (Fig. 12). The residues at the position of the catalytic tyrosine in ChdCs (arginine and glutamine) also appear to have no significant impact. The roles of the active site arginine and aspartate in DyPs have been widely discussed and investigated by mutational studies [85,89]. Recently it was demonstrated that the distal arginine in type B DyP of *Klebsiella pneumoniae* is highly important for the active site architecture, as the loop, discussed in section 2.4., collapses if the arginine is exchanged to alanine. This results in a complete reorientation of the distal aspartate, which has been shown to act as Lewis base in the active site, being responsible for deprotonation of hydrogen peroxide [31].

**Fig. 13:**
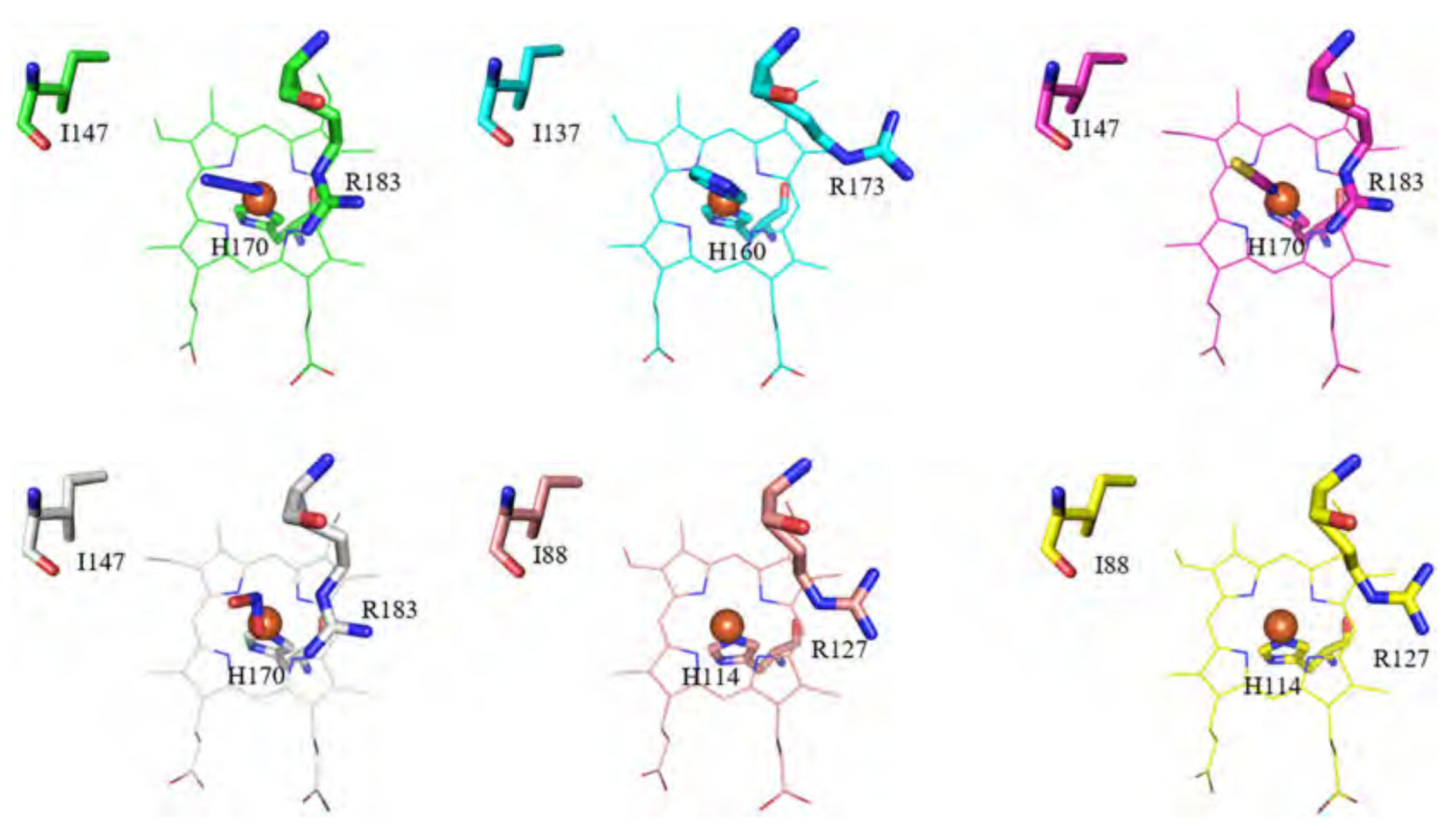
Distal side architecture of Clds. Relevant active site residues and heme molecules are shown in green (*Ms*Cld, 5A12), cyan (*Nd*Cld, 3NN1), magenta (*Ao*Cld, 2VXH), light grey (*Da*Cld 3Q09), salmon (*Nw*Cld, 3QPI) and yellow (*C*Cld, 5MAU). (For interpretation of the references to color in this figure legend, the reader is referred to the web version of this article.)

In Clds, the distal arginine is not interacting with a heme propionate group, as heme *b* is differently oriented (Fig. 6) and can adopt two distinct positions (Fig. 13). Among the discussed Cld structures, some have a distal heme ligand like azide (5A12), imidazole (3NN1), thiocyanate (2VXH), or nitrate (3Q09). The occupancy of the sixth position still does not seem to dictate the distal arginine either being in the “in” (*Ms*Cld, *Ao*Cld, *Da*Cld) or “out” (*Nd*Cld, *C*Cld, *Nw*Cld) position. The parameters deciding whether the arginine is “in” or “out” are not completely known to date. At the position of the catalytic tyrosine in ChdCs, a conserved isoleucine is found in Clds.

**Fig. 14:**
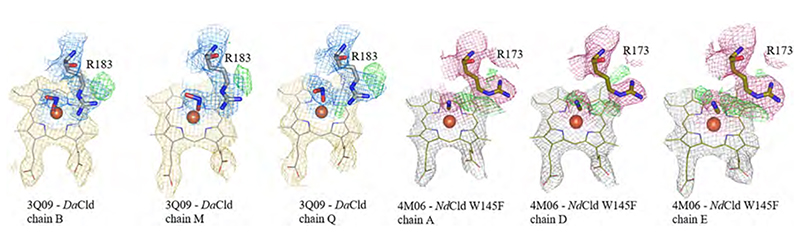
Distal side of *Da*Cld and *Nd*Cld W145 variant. Heme *b* is depicted as lines, the arginine as sticks. Electron density maps (2Fo-Fc) are shown in blue for distal side environment of *Da*Cld (arginine and nitrate) and in pink for *Nd*Cld W145F (arginine and cyanide), the densities of heme *b* in *Da*Cld are shown in yellow, and in grey for *Nd*Cld W145F. Unmodelled peaks of the difference map (2Fo-Fc) are depicted in green. (For interpretation of the references to color in this figure legend, the reader is referred to the web version of this article.)

The proposed role of the distal arginine, which is catalytically essential, is to keep the formed intermediate (either HOCl or OCl^.^) in place for a nucleophilic attack of the transient oxo-ferryl species. For this purpose, the distal arginine needs to be flexible, which is already indicated by examination of the available crystal structures (Fig. 13). The flexibility of the distal arginine was investigated in a molecular dynamics simulation study, which calculated not only the ferric and ferrous state, but also the Compound I state [92]. This study supports the hypothesis that the positively charged distal arginine is not responsible for binding of the negatively charged substrate chlorite, but is rather responsible to keep the transiently formed chlorine species in the active site. Due to photoreduction, the obtained crystal structures are all in their ferrous state, which has a significant impact to interatomic distances within the active site, as the redox state of the heme iron is crucial for ligand affinities [119]. Fig. 13 shows the arginine of each representative in one clear conformation, either “in” or “out”, which is not always the completely correct representation. In some structures, the flexibility of the distal arginine is evident from the experimentally derived electron densities of the active site. In Fig. 14 the active sites of some subunits of *Da*Cld (3Q09) and of the *Nd*Cld W145F (4M07) variant are presented with the model as it is deposited in the protein data bank (pdb) and the obtained electron density maps (2Fo-Fc) as well as the difference maps (Fo-Fc). While the distal arginine in *Da*Cld was modelled in an “in” position and the arginine of the *Nd*Cld W145F variant in an “out” position, it is evident from the analysis of the electron densities that also the respective other orientation is present. In both cases a model containing both conformations would be more accurate (Fig. 14).

This emphasizes that care has to be taken when structures are refined, as slightly imprecise modelling and refinement of the active site can have significant implications on functional interpretation. These inaccuracies are not reflected in the overall statistics of the structure refinement and enzymologists tend to work with the deposited models of the respective structures and do not evaluate electron densities in detail. Ideally, non-photoreduced structures of the ferric state should be available, as for *Kp*DyP (multi-crystal approach) (6RQY, 6RPE) [119] or *C*Cld (neutron structure) [58].

**Fig. 15:**
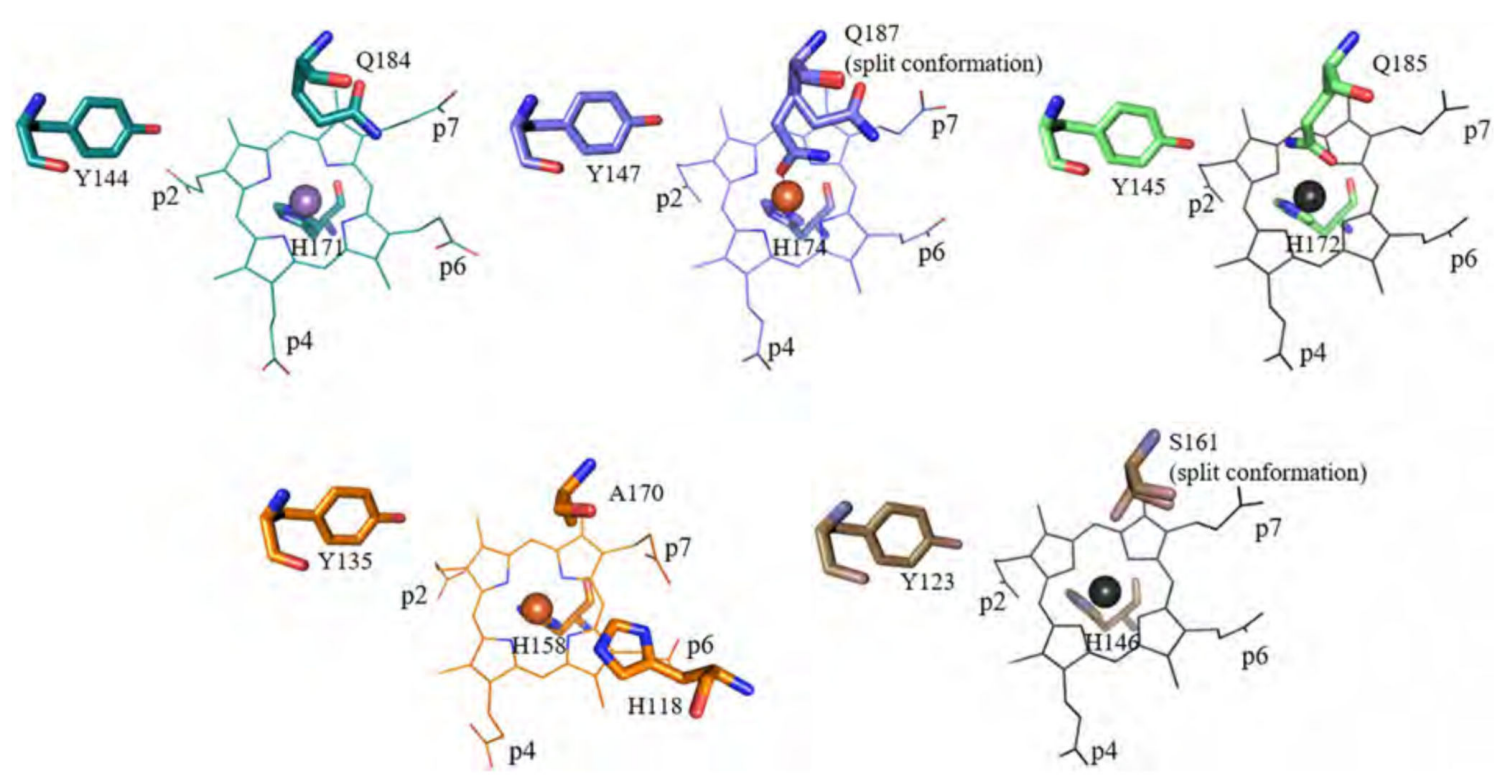
Distal side architecture of ChdCs. The colour code is the following: turquoise (*Gs*ChdC, 5T2K), blue (*Lm*ChdC, 6FXJ), lightgreen (*Tt*ChdC, 1VDH), orange (*Cd*ChdC, 6XUC), dark orange (*Ta*ChdC, 3DTZ). The coproheme of the *Lm*ChdC structure was placed in apo-structures of *Ta*ChdC and *Tt*ChdC in black for better orientation. (For interpretation of the references to color in this figure legend, the reader is referred to the web version of this article.)

The active site of ChdC shows the highest degree of heterogeneity of all three discussed protein classes. The available structures (Fig. 15) are limited in number but emphasize that (i) the distal side residues vary from one phylogenetic clade to the other, that (ii) actinobacterial but not firmicute ChdCs have a histidine as distal base, that (iii) the porphyrin can be present in more than one orientation within the ChdCs’ active site [6,7,20,46]. In firmicute ChdCs, a glutamine is found on the distal side, which can serve as a six coordinated low-spin (6cLS) ligand and has a regulatory function as was demonstrated by in-solution resonance Raman studies [93]. When the glutamine was exchanged to alanine, a higher amount of hydrogen peroxide was needed for complete decarboxylation of coproheme. Furthermore, in the presented structure glutamine exhibits a double conformation (Fig. 15), indicating flexibility. This was confirmed by resonance Raman spectroscopy, where carbon monoxide ligand binding was tested to the ferrous protein and an open and closed cavity was detected for the Q187A variant and the wild-type protein, respectively [93]. In firmicute ChdCs, a conserved methionine, positioned between p2 and p4, contributes to the stability of the active site architecture. It establishes a sulfonium-ion linkage, similar as in human myeloperoxidase [94–98] to the formed vinyl group of the product heme *b*, when hydrogen peroxide is present in excess [93]. The integrity of the active site and the maintenance of the wild-type like spin-state of the heme iron is further guaranteed by the hydrogen bonding network spanning from residues interacting with p2 (arginine, serine) to residues interacting with p4 (lysine) [91]. Disturbance of this H-bonding network leads to a collapse of the active site, as was demonstrated by electronic circular dichroism spectroscopy [91]. The catalytic tyrosine adopts the same orientation in different ChdCs, in presence and absence of coproheme (Fig. 9). In the structure of *Cd*ChdC, a distal histidine is found at the position of the distal aspartate in DyPs. This histidine is also part of the flexible loop described above.

## 3 Catalytic reaction mechanisms of porphyrin binding α + β barrel proteins

### 3.1 Chlorite dismutase

**Scheme 1:**
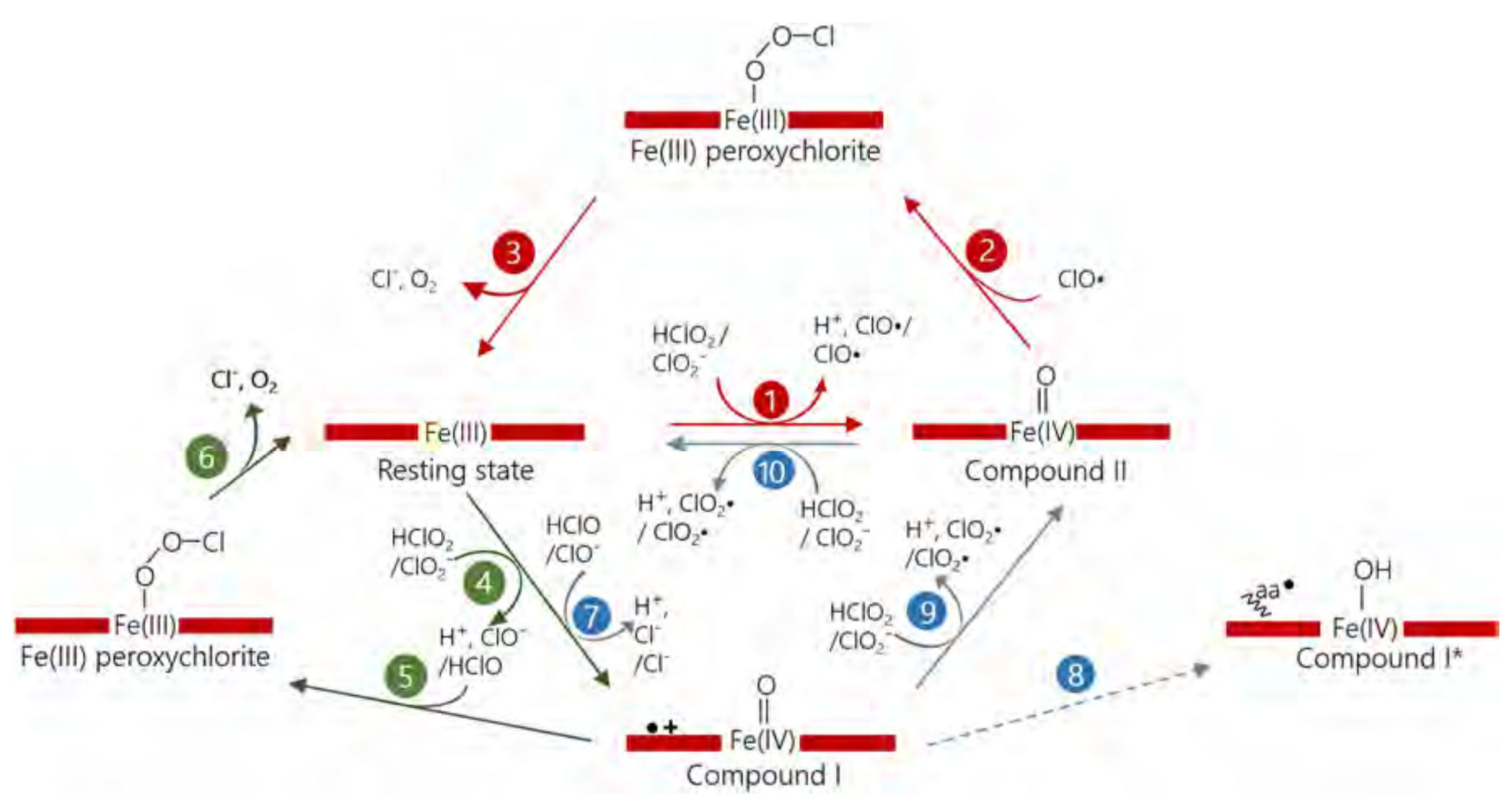
Homolytic cleavage (highlighted in red) heterolytic cleavage (highlighted in green) of chlorite and side reactions (highlighted in blue) of ferric *C*Cld with chlorite.

Clds are oxidoreductases which reduce chlorite (ClO_2_^−^) to chloride (Cl^−^) and produce dioxygen (O_2_); a covalent oxygen-oxygen bond is formed. Clds are only the second enzymatic system found to be capable of O–O bond formation besides the manganese containing water splitting complex of photosystem II [99]. The observation that the substrate (ClO_2_^−^) to product (O_2_) ratio is 1:1 leads to the conclusion that both oxygen atoms of the product dioxygen evolve from the one chlorite molecule [12]. Indeed, chlorite was identified as the sole source of dioxygen by O^18^-labeling studies combined with mass spectrometry [100]. Consequently, an initially proposed reaction mechanism indicating a 2:1 chlorite to dioxygen ratio, which did not use Cld but a water soluble iron porphyrin, cannot be possible [101]. In this case, chlorite (ClO_2_^−^) would be reduced to hypochlorite (ClO^−^) and a Compound I species would be formed, a second chlorite molecule would undergo the same reduction and dioxygen would be produced, but no chloride, as two equivalents hypochlorite accumulate. To be in line with the determined 1:1 stoichiometry, the O–Cl bond of chlorite needs to be cleaved either homolytically (reaction 1, 2 and 3) or heterolytically (reaction 4,5,6). Heterolytic cleavage, as in the previously proposed model [100], would yield hypochlorite and Compound I (reaction 4) followed by a rebound mechanism to yield chloride and dioxygen (reaction 5 and 6 analogous to reactions 2 and 3 in Scheme 1). In case of a homolytic cleavage, the intermediates have to be OCl^.^and Compound II [102].

Many studies aimed at identifying the relevant redox intermediate of chlorite dismutases during catalysis [100,103,104]. For a long time, experimental studies were favoring, but not proving, a heterolytic cleavage of the O–Cl bond as a first reaction step. This was based on spectroscopic studies which indicated Compound I formation with chlorite [100] or peroxyacetic acid [103]. Also, inhibition of chlorite dismutase was investigated in detail and indicated that hypochlorite escapes the active site during turnover and oxidatively inactivates the enzyme [92,104]. This is only possible if a heterolytic cleavage occurs (reaction 4, 5 and 6). Computational studies on the other hand were favoring homolytic cleavage (reaction 1 followed by reactions 2 and 3), as the energy barriers for this reaction path are significantly lower than for the heterolytic model [102,105].

In a recent study on a dimeric chlorite dismutase (*C*Cld), the complexity of the enzymatic reduction of chlorite was studied in detail by in-solution spectroscopic approaches (stopped-flow UV–vis, RR) and crystallographic studies (X-ray and neutron diffraction) [58]. Even though the pH profile of the reaction is difficult to interpret, the fact that Compound I formation but not oxygen generation was observed with hypochlorite as substrate (reaction 7), excludes the possibility that Compound I and hypochlorite react in a rebound mechanism as a second reaction step. On the other hand, spectroscopic signatures during turnover with chlorite are indicative of a Compound II [58]. Nevertheless, under acidic conditions (the pH optimum of the chlorite reduction) there is also a spectral evidence for Compound I formation. This may suggest the existence of another reaction pathway (side reactions in Scheme 1, reactions, 7 and 8) that utilizes Compound I as an intermediate [58] and possibly yields Compound I* (reaction 8) or Compound II (reaction 9) and the ferric enzyme (reaction 10) under formation of chlorine dioxide. In summary, the most recently proposed reaction mechanism includes homolytic cleavage of the O–Cl bond, resulting in an OCl^.^ radical and Compound II, followed by nucleophilic attack to the ferryl oxygen resulting in release of O_2_ and Cl^−^ (Scheme 1, reactions 1–3) but cannot unequivocally rule out the heterolytic cleavage.

The properties of the catalytically relevant amino acid residues of the active site are important in order to completely understand the catalytic mechanism. Sequence analysis and first crystal structures provided a preliminary insight into the active site cavity and the heme *b* environment [6,7,14,57,106,107]. As already discussed, the heme cofactor is coordinated by a proximal histidine and the hydrophobic distal side is dominated by an arginine residue. This arginine was exchanged by several amino acid residues (alanine, lysine, glutamate, glutamine) and the thorough investigation of these variants proved that the distal arginine is catalytically highly important, albeit not completely essential [7,80,81]. The mechanistic role of the distal arginine is still under discussion. Analyses of steady-state and pre-steady-state kinetics with chlorite, but also with ligands (e.g. cyanide) showed that the initial binding of the anionic substrate chlorite (p*^K^*_a_ = 1.72) [108] was not influenced as much as the turnover number [7,80,81]. This is in agreement with computational molecular dynamics simulations [92]. Likewise, the reason for the pronounced pH dependence of chlorite conversion (pH optimum of Clds is in the acidic region below pH 5.5 [46]) is still elusive. The protonation state of the distal arginine was at one point discussed to be crucial, as one report suggested that the distal environment lowers the p*^K^*_a_ of the arginine in *Da*Cld to 6.5 [13], whereas the alkaline transitions for other Cld representatives were at p*^K^*_a_ values between 8.1 and 8.7 [58,109,110]. The reported p*^K^*_a_ value of hypochlorite (HOCl/OCl^−^) is at 7.53 which, if altered slightly by the protein environment, would be closer to the transition towards the pH optimum for catalysis in Clds. Nevertheless, the neutron structure of *C*Cld showed a protonated distal arginine at pH 9.0 [58].

These data together with data on enzyme inactivation during turnover of wild-type Clds and arginine variants indicate that the distal arginine is important for the second reaction step (reaction 2, homolytic cleavage, reaction 5 heterolytic cleavage), which includes the rebound mechanism and the recombination of the oxygen molecules to form dioxygen. The currently proposed role of the catalytic arginine is to be a gate keeper and coordinate the intermediate chlorine species (either OCl^.^ or hypochlorite) in the active site [58,92,104]. Above, we already showed that the distal arginine is flexible; this further supports a regulatory coordinating role during catalysis (Figs. 13, 14).

### 3.2 Dye decolorizing peroxidases

For DyPs it is challenging to describe a universal catalytic reaction mechanism valid for all phylogenetic clades. The diversity of possible substrates and the uncertainty about the respective physiological roles make it almost impossible to discuss them as an independent group of enzymes (see sections above). Nevertheless, all DyPs initially react with hydrogen peroxide, which classifies them as peroxidases. The active site architecture of DyPs is almost exclusively described by a proximal iron-coordinating histidine, a distal aspartate and a distal arginine (Figs. 10, 11). In a few cases, the distal aspartate is replaced by a glutamate, which results in significantly lower activities [44]. Mutational studies, targeting the distal residues in DyPs, proved the importance of both residues, but different roles have been proposed concerning the movement of the aspartate and the essentiality of the arginine [31,66,85,89]. The current hypothesis is that the distal aspartate is responsible for deprotonation of the incoming hydrogen peroxide, which enables subsequent heterolytic cleavage, and that the arginine has a stabilizing task in order to maintain the active site architecture [31]. Interestingly the determined pH maxima of any DyP are always below pH 5.0 and are sometimes found to be in extreme acidic conditions [26,34,39,65,67,73,75,111].

**Scheme 2:**
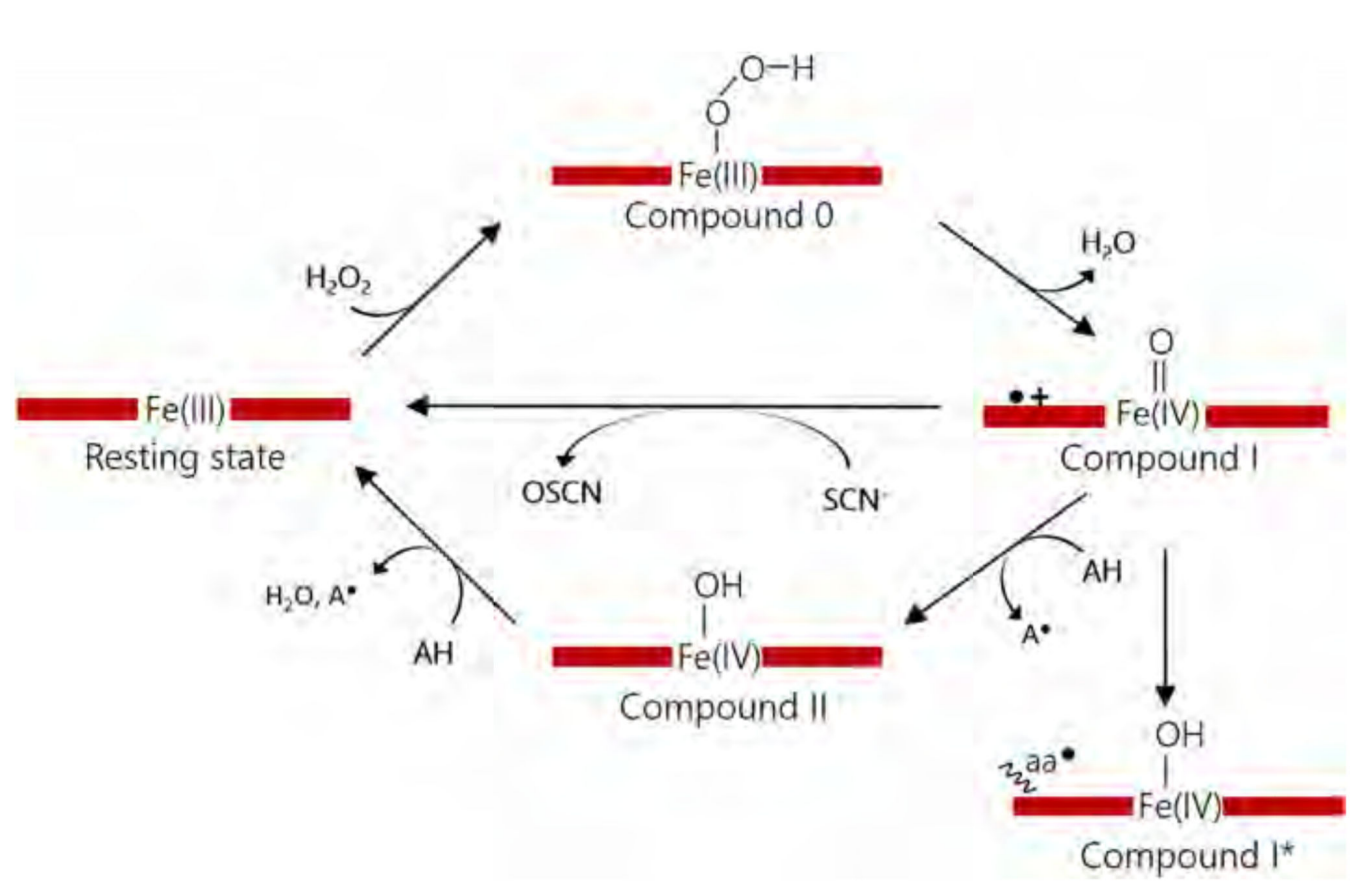
Proposed reaction mechanism of DyPs

Heterolytic cleavage of the O–O bond of deprotonated hydrogen peroxide leads to the formation of Compound I [Fe(IV) = O Por^.+^] and H_2_O (Scheme 2). This first reaction step is also commonly performed by other peroxidases [47,112]. The formation of Compound I can be followed very nicely in type B DyPs by spectroscopic techniques (UV–vis, EPR) [31,39,73,113,114]. The Compound I intermediate is highly stable and does not react easily with a variety of substrates (electron donors), which could reduce Compound I to Compound II [Fe(IV)-OH Por] via a one-electron reduction or to the ferric resting state via a two-electron reduction. Due to the lack of a physiological substrate and the fact that Compound I is highly stable, the underlying reaction mechanism was intensely debated [73,113]. Formation of Compound II was not observed directly by spectroscopic techniques until recently, when stopped-flow spectroscopic studies trapped the Compound II intermediate with serotonin as one-electron donor [31]. This observation confirms that type B DyPs follow a classical peroxidase cycle, which is a two-step mechanism as first suggested by Poulos and Kraut (Scheme 2) [115,116].

Type A DyPs are supposed to play a role in bacterial iron metabolism and are postulated to oxidize Fe^2+^ to Fe^3+^ [5,32]. In pre-steady-state kinetics studies, reaction with hydrogen peroxide yields a spectrum probably displaying Compound I* [Fe(IV)-OH Por…aa^.^], which is isoelectronic to Compound II [39]. In type C/D DyPs, Compound I is very short-lived and directly followed by formation of Compound I* [Fe (IV)-OH Por…aa^.^] (Scheme 2), since the porphyryl radical rapidly migrates to an aromatic amino acid residue in close proximity to the heme. This transition was nicely demonstrated for DyP from *Auricularia auricula-judae* by spectroscopic and computational methods [67]. This radical migration is necessary for long range electron transfer from the heme to surface exposed aromatic amino acids (tyrosines), which interact with large bulky substrates and subsequently oxidize them [29,67,111]. Type C/D DyPs are of high interest for potential biotechnological applications due to their broad substrate range and high pH stability in the acidic region. The reason for the observed pH dependence of steady-state reactions is not completely understood today and strongly depends on the used substrates.

### 3.3 Coproheme decarboxylases

ChdCs catalyze the ultimate step of the coproporphyrin-dependent heme biosynthesis pathway by decarboxylating propionate groups at positions 2 and 4 of iron coproporphyrin III (coproheme) to yield heme *b* (iron protoporphyrin IX) [16,20]. The mode of action includes a transiently produced three-propionate intermediate (monovinyl, monopropionate deuteroheme - MMD; sometimes also referred to as harderoheme) [16,19,61,117]. The reaction requires an oxidant, which most probably is hydrogen peroxide, but also peroxyacetic acid and flavin mononucleotide (FMN) were shown able to initiate the reaction [17,19,61]. The general catalytic efficiency of coproheme conversion is rather low in ChdCs from Firmicutes (approximately 10^2^ M^−1^ s^−1^), and a 2:1 (oxidant:substrate) stoichiometry was determined [61].

**Scheme 3:**
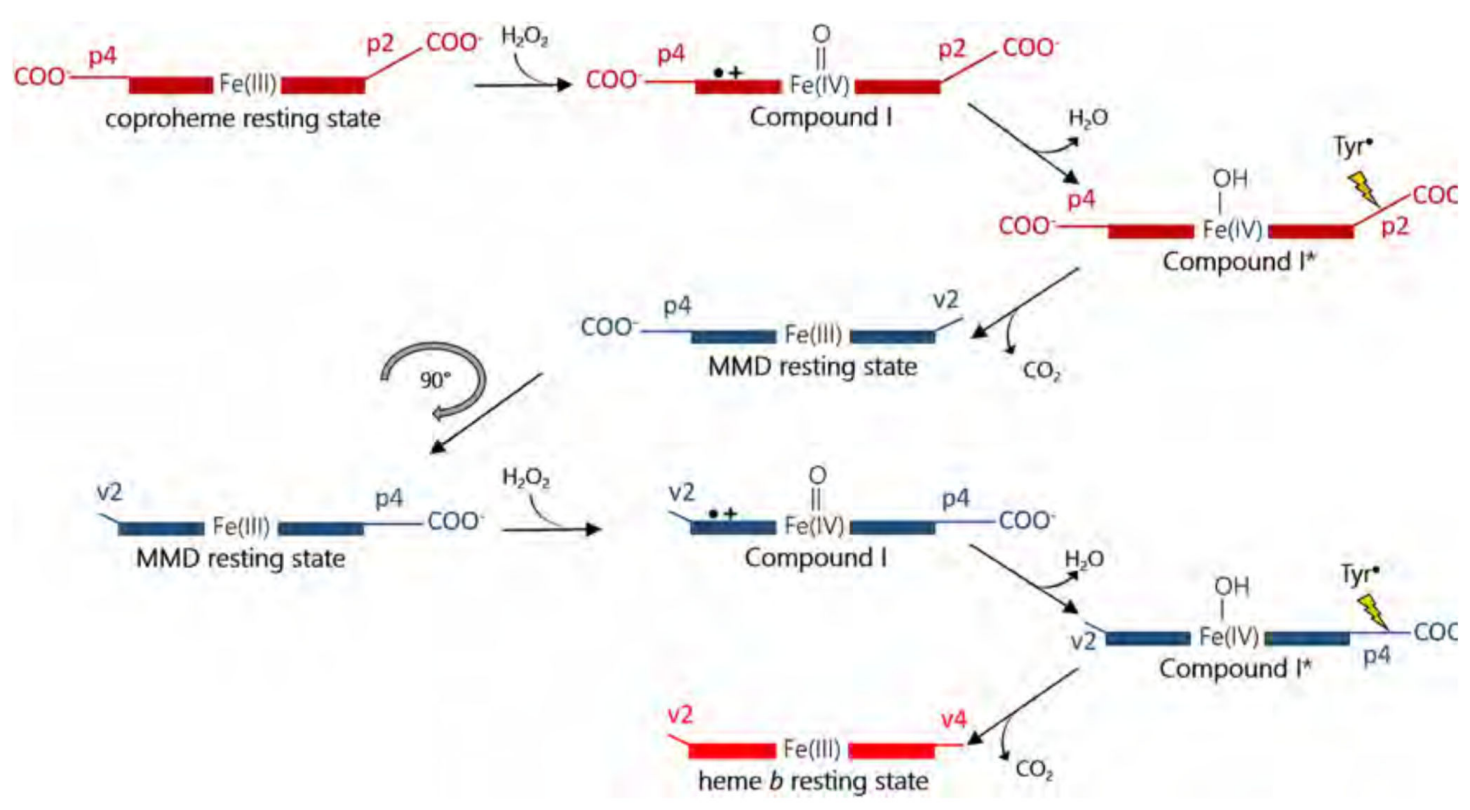
Proposed reaction mechanism of Coproheme decarboxylases. The first decarboxylation step is initiated by oxidation of resting state ChdC by hydrogen peroxide to form Compound I which is rapidly converted to Compound I*. The propionate at position 2 is decarboxylated and the resulting three-propionate monovinyl monopropionate deuteroheme (MMD) intermediate is rotated by 90°, thereby bringing p4 close to the tyrosine. A second hydrogen peroxide molecule is necessary for decarboxylation of p4.

A tyrosine residue was identified to be essential for catalysis (Fig. 15, Scheme 3). This tyrosine is located in close proximity to the propionate at position 2 (p2) in the coproheme-resting state [59,91]. The role of the tyrosine was unraveled by electron paramagnetic resonance spectroscopic studies [90] and spin trapping experiments [62]. Ultimately, the tyrosine proved to be the radical site responsible for the attack to the β-carbon of the propionate group. This proton-coupled electron transfer mechanism is also supported by density functional theory calculations [118]. The positively charged residue interacting with p4 in the resting state (lysine in Firmicutes, arginine in Actino-bacteria) is also crucial for turnover; exchange of this residue leads to severely hampered activity and incomplete heme *b* production [59,91].

The fact that two propionates are cleaved off led to three possible reaction mechanisms. One proposal required the enzyme harboring two active sites; one being the described tyrosine and another one would have to be a radical site in proximity to p4. This reaction mechanism can be ruled out as only the tyrosine residue close to p2 is responsible for both decarboxylation reactions [90]. Secondly, a long range electron transfer from the tyrosine residue to p4 would be possible but was deemed unlikely by computational analysis [62]. The third possibility involves a rearrangement of the monovinyl, monopropionate deuteroheme (MMD) intermediate, which would bring p4 into the location previously occupied by p^2^ (Scheme 3). This rearrangement was observed in X-ray crystal structures of the actinobacterial ChdC from *Corynebacterium diphteriae* [64] and firmicute ChdC from *Listeria monocytogenes* and, most convincingly, also in solution by extensive resonance Raman spectroscopic studies [62]. It is unclear to date whether the substrate rotates within the active site or the three-propionate intermediate is released and rebinds in a different orientation. Furthermore, the orientation of the product heme *b*, as well as the exact mechanism of product transfer to heme *b* receiving apo-enzymes is still unknown. In excess of hydrogen peroxide, the product heme *b* is covalently linked to a methionine residue in the active site [93], most probably forming a sulfonium-ion linkage similar to the one established in human myeloperoxidase [94–96]. This crosslinking of the heme *b* group is most likely an *in vitro* artefact, as hydrogen peroxide concentrations needed for the cross-linking are above physiological levels.

Many open questions concerning the details of the redox reaction remain unanswered. As a fact, ChdC is able to form Compound I, however, in Firmicutes ChdCs the two-electron deficient species could only be shown with chlorite (p*^K^*_a_ = 1.72) as oxidant, which is clearly not the physiological substrate [62]. Hydrogen peroxide as an oxidant needs to be activated by deprotonation prior to formation of Compound I. However, firmicute ChdCs lack a distal base in the active site, consequently no Compound I formation with hydrogen peroxide could be observed. However, the presence of a Compound I*, manifested in the formation of the tyrosine radical, indicates that a Compound I must be formed initially. The structure of *Cd*ChdC (actinobacterial enzyme) revealed a distal histidine, located on the flexible linker loop that connects the two ferredoxin-like domains, which was shown to be responsible for hydrogen peroxide deprotonation. The overall reaction of coproheme conversion in actinobacterial ChdCs is faster and more efficient compared to firmicute ChdCs. In addition, a Compound I was trapped in *Cd*ChdC when hydrogen peroxide was used [64].

## 4 Future perspectives

In-depth knowledge of the electronic reaction mechanism of the discussed enzymes (Clds, DyPs, ChdCs) is essential to be able to exploit their catalytic portfolio for biotechnological and biomedical purposes. Clds are of interest because their remarkable ability to catalyze the formation of an oxygen-oxygen bond and further due to their ability to detoxify chlorite, which is a common environmental pollutant, being abundant in many ground water reserves. Therefore, Clds are promising candidates for detoxification of polluted environments and engineered Cld variants will potentially play a significant role for bioremediation in the future.

As is evident from the chapters above, DyPs can oxidize a myriad of compounds, being promising candidates to carry out biotechnological tasks. These tasks can reach from lignin degradation to environmental-friendly bleaching of dyes. DyPs may also be used as a starting scaffold for protein engineering approaches, which aim to select and improve a certain specialized activity.

ChdCs have been identified as a promising target for the development of highly needed novel antibiotic therapeutic substances. Inhibition of ChdCs in pathogenic monoderm bacteria is a potent way to kill the pathogen, as heme biosynthesis will be interrupted, which is essential for most bacteria. ChdC does not have a homologous mammalian counterpart, which is a perfect precondition. The quest for a mechanism based inhibitor is one of the major tasks for future research projects.

## Abbreviations

Clds: chlorite dismutases;
ChdCs: coproheme decarboxylases
DyPs: dye-decolorizing peroxidases
DyPA: dye-decolorizing peroxidase from type A
DyPB: dye-decolorizing peroxidase from type B
EfeBs: DyPA from *Escherichia coli*
DyPC/D: dye-decolorizing peroxidase from type C and D
OxdA: aldoxime dehydratases
SCOP: structural classification of proteins
IsdGs: heme degrading enzymes
*Ms*Cld: chlorite dismutases from *Magnetospirillum sp*.
*Nd*Cld: chlorite dismutase from *Nitrospira defluvii*
*Ao*Cld: chlorite dismutase from *Azospira oryzae*
*C*Cld: chlorite dismutase from *Cyanothece sp.*
*Nw*Cld: chlorite dismutase from *Nitrobacter winogradskyi*
*Da*Cld: chlorite dismutase from *Dechloromonas aromatica*
*Kp*DyP: dye-decolorizing peroxidases from *Klebsiella pneumoniae*
*Aau*DyP: dyedecolorizing peroxidases from *Auricularia auricula-judae*
*Ec*DyP: dye-decolorizing peroxidases from *Escherichia coli*
*Ba*DyP: dye-decolorizing peroxidases from *Bjerkandera adusta*
*Gs*ChdC: coproheme decarboxylase from *Geobacillus stearothermophilus*
*LmC*hdC: coproheme decarboxylase from *Listeria monocytogenes*
*Cd*ChdC: coproheme decarboxylase from *Corynebacterium diphteriae*
*Tt*ChdC: coproheme decarboxylase from *Thermus thermophilus*
*Ta*ChdC: coproheme decarboxylase from *Thermoplasma acidophilum*
*Sa*ChdC: coproheme decarboxylase from *Staphylococcus aureus*
propionate 2: p2
propionate 4: p4
propionate 6: p6
propionate 7: p7
